# Horizontal Gene Transfer Can Rescue Prokaryotes from Muller’s Ratchet: Benefit of DNA from Dead Cells and Population Subdivision

**DOI:** 10.1534/g3.113.009845

**Published:** 2013-12-17

**Authors:** Nobuto Takeuchi, Kunihiko Kaneko, Eugene V. Koonin

**Affiliations:** * National Center for Biotechnology Information, National Library of Medicine, National Institutes of Health, Bethesda, Maryland 20894; † Department of Basic Science, Graduate School of Arts and Sciences, University of Tokyo, Tokyo 153-8902, Japan

**Keywords:** environmental DNA, evolution of transformation, competence, structured population, soil bacteria

## Abstract

Horizontal gene transfer (HGT) is a major factor in the evolution of prokaryotes. An intriguing question is whether HGT is maintained during evolution of prokaryotes owing to its adaptive value or is a byproduct of selection driven by other factors such as consumption of extracellular DNA (eDNA) as a nutrient. One hypothesis posits that HGT can restore genes inactivated by mutations and thereby prevent stochastic, irreversible deterioration of genomes in finite populations known as Muller’s ratchet. To examine this hypothesis, we developed a population genetic model of prokaryotes undergoing HGT via homologous recombination. Analysis of this model indicates that HGT can prevent the operation of Muller’s ratchet even when the source of transferred genes is eDNA that comes from dead cells and on average carries more deleterious mutations than the DNA of recipient live cells. Moreover, if HGT is sufficiently frequent and eDNA diffusion sufficiently rapid, a subdivided population is shown to be more resistant to Muller’s ratchet than an undivided population of an equal overall size. Thus, to maintain genomic information in the face of Muller’s ratchet, it is more advantageous to partition individuals into multiple subpopulations and let them “cross-reference” each other’s genetic information through HGT than to collect all individuals in one population and thereby maximize the efficacy of natural selection. Taken together, the results suggest that HGT could be an important condition for the long-term maintenance of genomic information in prokaryotes through the prevention of Muller’s ratchet.

Evolutionary advantages of recombination have been extensively considered, primarily in relation to the evolution of sex in eukaryotes (Kondrashov 1993; Maynard Smith 1978, 1998). However, advances in genome sequence analyses have revealed that many prokaryotes, whose mode of reproduction is asexual (*i.e.*, no meiosis or syngamy), frequently undergo genetic recombination, the process known as horizontal gene transfer (HGT; Feil *et al.* 2000, 2001; Ochman *et al.* 2000; Koonin *et al.* 2001; Gogarten *et al.* 2002; Vos and Didelot 2009; Yahara *et al.* 2012). Numerous studies indicate that HGT has been instrumental in producing functionally consequential genomic changes throughout the evolution of prokaryotes (Lerat *et al.* 2005; Dagan *et al.* 2008; Treangen and Rocha 2011). For example, HGT is implicated in the evolution of various functional systems and specific adaptations such as various metabolic pathways (Pál *et al.* 2005; Maslov *et al.* 2009), oxygenic photosynthesis (Mulkidjanian *et al.* 2006), thermal resistance (Aravind *et al.* 1998; Brochier-Armanet and Forterre 2007), antibiotic resistance (Blahna *et al.* 2006), pathogenicity (Kelly *et al.* 2009), and others. Moreover, it has been shown that HGT can substantially impact microbial community development (Burke *et al.* 2011) and ecological diversification (Shapiro *et al.* 2012). The prevalence and significance of recombination in prokaryotes as well as in eukaryotes point to the crucial importance of recombination for the evolution of life in general (Komatsu 1980).

HGT in prokaryotes occurs though three basic mechanisms: conjugation, transduction, and transformation (Levin 1988; Thomas and Nielsen 2005; Johnsborg *et al.* 2007). Conjugation and transduction involve transfer of prokaryotic DNA between cells mediated by infectious agents such as viruses and conjugative plasmids, respectively. Because genes involved in these mechanisms reside in the genomes of mobile genetic elements, transduction and conjugation can be considered side effects of the selfish propagation of these infectious agents (Redfield 2001).

Transformation, by contrast, is a bacterium-programmed mechanism of HGT that is mediated by natural competence systems encoded in the genomes of many bacteria (Lorenz and Wackernagel 1994; Claverys *et al.* 2009). Through transformation, a cell absorbs and integrates extracellular DNA (eDNA) into its chromosome. Although transformation might be viewed as a side effect of metabolic consumption of eDNA (Redfield 2001), there is increasing evidence, at least in several bacteria, that absorbed eDNA serves as the source of information rather than or at least in addition to being a source of nutrients (Johnsborg *et al.* 2007; Claverys *et al.* 2009; Zafra *et al.* 2012; Johnston *et al.* 2013). Such evidence includes, *e.g.*, the existence of competence-induced proteins (*e.g.*, DprA in *Streptococcus pneumoniae*) that protect absorbed eDNA from degradation by intracellular nucleases (for other lines of evidence, see the references cited above).

Besides transformation, there is another mechanism of HGT called gene transfer agents (GTAs), which is encoded in the genomes of many prokaryotes (Lang and Beatty 2007; McDaniel *et al.* 2010; Lang *et al.* 2012). The GTAs are defective virus particles that encapsulate apparently random fragments of the host DNA (rather than the genes encoding the GTA proteins) and mediate the transfer of these fragments into other prokaryotic cells. At face value, the GTAs appear to function as dedicated vehicles for HGT. The existence of these apparently prokaryote-programmed mechanisms of HGT suggests an intriguing possibility that these mechanisms have been maintained by selection in prokaryotes owing to evolutionary advantages offered by HGT—a situation akin to what is currently believed for sexual recombination in eukaryotes (Michod and Levin 1988; Kondrashov 1993; Keightley and Otto 2006; see Bernstein *et al.* 1988 and Redfield 2001 for counteracting views).

What selective advantages could HGT offer to prokaryotes? To address this question, we asked whether an evolutionary advantage of recombination that has been originally suggested for eukaryotes could be extended to the case of HGT in prokaryotes. In particular, we hypothesized that HGT could restore genes that are deleted or inactivated by mutations, thereby preventing the stochastic, irreversible deterioration of genomes in finite populations known as Muller’s ratchet (Muller 1964; Felsenstein 1974; Pamilo *et al.* 1987; Bell 1988; Charlesworth *et al.* 1993; for other types of advantages, see, *e.g.*, Redfield *et al.* 1997; Cooper 2007; Levin and Cornejo 2009; Vos 2009; Wylie *et al.* 2010). This hypothesis is consistent with the observation of extreme genome reduction and accelerated protein evolution in intracellular bacterial parasites and symbionts (Moran 1996; Novichkov *et al.* 2009; Merhej and Raoult 2011; McCutcheon and Moran 2012). Because of continual bottlenecks and sequestration within the host organisms, these bacteria are likely to have small effective population sizes and be effectively isolated from HGT between populations in different hosts. Under these conditions, the impact of Muller’s ratchet increases and could lead to genome reduction, accelerated protein evolution, and possibly eventual extinction (Lynch *et al.* 1993; Merhej and Raoult 2011). Therefore, HGT might be an important requirement for the long-term maintenance of genomic information in prokaryotes (Koonin 2011).

However, HGT in prokaryotes differs from eukaryotic recombination both in quantity and in quality. Quantitatively, HGT is relatively less frequent and affects a far smaller portion of chromosomes per recombination event than eukaryotic recombination that is inextricably linked to sexual reproduction (Dubnau 1999; Feil *et al.* 2000). Qualitatively, whereas eukaryotic recombination is a direct exchange of DNA sequences between haploid genomes inherited from the two parents, HGT in prokaryotes, in particular, transformation is an indirect exchange of DNA sequences between cells via an eDNA pool (Redfield 1988). The sources of eDNA can be diverse, but the most common one is likely to be the debris of dead conspecific cells. If the death rate of a cell increases with the number of deleterious mutations in the genome, the genomes of dead cells on average carry more deleterious mutations than those of living cells (Redfield *et al.* 1997). Consequently, transformation on average is expected to introduce more deleterious mutations than it would remove. In view of these differences between eukaryotic recombination and HGT in prokaryotes, which potentially nullify the hypothetical benefit of HGT, we sought to assess the net effect of HGT on the operation of Muller’s ratchet by performing stochastic population genetic simulations.

We analyzed a simple evolutionary model of finite prokaryotic populations undergoing HGT. Our model is based on the infinite population model studied by Redfield (1988) with several extensions (see the section *Materials and Methods*). The model was analyzed under the assumption that is most unfavorable to the hypothesized advantage of HGT, namely, that variation in fitness is attributed solely to variation in death rates, and the fittest individuals do not contribute their genomes to the eDNA pool. The results show that, even under this assumption, HGT can prevent the operation of Muller’s ratchet by causing a continual generation of the least-loaded class (*i.e.*, genomes with the least number of deleterious mutations) from more-loaded classes. Moreover, the prevention of Muller’s ratchet by HGT is substantially more efficient in a subdivided population than in a panmictic population of an equal overall size, provided that the diffusion of eDNA is sufficiently rapid.

## Materials and Methods

Definitions for the symbols used in this article can be found in Table 1.

**Table 1:** List of symbols

Symbol	Description
*N*	Total population size
*U*	Mutation rate per genome per generation
*s*	Fitness effect of a mutation (= 0.01)
*r*	Recombination (HGT) rate per genome per generation
*l*	Number of loci in a genome
*d*_eDNA_	Turnover rate of eDNA per generation
*D*_pop_	Population migration rate per generation
*D*_eDNA_	eDNA diffusion rate per generation
*n*_i_	Population size of cells with *i* mutations
*m*_LLC_	Number of mutations in the least-loaded class
*m*_fix_	Number of mutations fixed in the population
${\bar{n}}_{m_{\text{LLC}}}$	$Ne^{-U/s}$: the steady-state population size of the least-loaded class
$H_{m_{\text{LLC}}}$	Average gene diversity of the least-loaded class
$H_{m_{\text{LLC}}-1}$	Average gene diversity of one but the least-loaded class
*q*_eDNA_	eDNA quality: the potential of the eDNA pool to remove deleterious mutations in the genomes of the least-loaded class
*t*_eDNA_	eDNA toxicity: the potential of the eDNA pool to introduce deleterious mutations in the genomes of the least-loaded class
*r*_01_	Probability of HGT introducing mutations in the simplified Fisher-Wright model
*r*_10_	Probability of HGT decreasing mutations in the simplified Fisher-Wright model

HGT, horizontal gene transfer; eDNA, extracellular DNA .

### General description of the model

The model consists of two components: a population of prokaryotic cells and a pool of eDNA. The population undergoes a standard mutation-selection process (Wright-Fisher model) as well as HGT, through which cells acquire alleles randomly drawn from the eDNA pool. The eDNA pool gains input from the population due to natural cell death and also undergoes spontaneous decay. The details of each of these processes are described in the sections to follow.

### Selection

The population dynamics was modeled as the discrete-generation Wright-Fisher process with fixed population size *N*. The genome was modeled as *l* loci (haploid), each representing a genomic segment that could be replaced by one recombination event (Wylie *et al.* 2010). The typical lengths of such segments are known to be on the order of 10 kb in several bacteria (Dubnau 1999; Feil *et al.* 2000), and the lengths of bacterial genomes are on the order of 1 Mb. Accordingly, the value of *l* was set to 100 unless otherwise stated. Each locus was assigned one of the infinite number of possible alleles, each of which was represented by a pair of integers: one integer indicating the locus and the other one indicating the number of deleterious mutations. All deleterious mutations were assumed to cause an equal fitness effect *s* without epistasis; thus, the relative fitness *f*_i_ of a genome with *i* mutations is *f*_i_ = (1 − s)*^i^*. This simplified model was adopted so that the effect of HGT on Muller’s ratchet could be assessed without confounding the additional effects caused by epistasis (Redfield *et al.* 1997). The speed of Muller’s ratchet is known to depend on the two combinations of parameters, *U*/*s* and *sN* (Neher and Shraiman 2012). Thus, in this study, the value of *s* was set to 0.01, and the values of *U* and *N* (more specifically, *sN* exp(−*U*/*s*) (denoted as $s{\bar{n}}_{m_{\text{LLC}}}$) and *N*) were varied to explore the behavior of the model.

### Mutation

In the model, each genome acquired the Poisson distributed number of new deleterious mutations with the mean *U* per generation. Beneficial mutations were ignored because we were concerned with the possible deterioration or maintenance of initially well-adapted genomes, for which the frequency of beneficial mutations is likely to be rare (Maynard Smith 1978; Charlesworth *et al.* 1993; cf. Goyal *et al.* 2012).

### Horizontal gene transfer

HGT generally can be categorized into homologous recombination and illegitimate (*i.e.*, nonhomologous) recombination. Because homologous recombination is much more frequent than nonhomologous recombination (Hulter and Wackernagel 2008; Brigulla and Wackernagel 2010), only the former was included in the model. In each genome, homologous recombination replaced the Poisson distributed number of alleles with mean *r* per generation per genome with alleles randomly drawn from the eDNA pool (after the replacement the original alleles were discarded).

### Dynamics of the eDNA pool

The frequency of homologous recombination decreases exponentially with the divergence between DNA sequences (Watt *et al.* 1985; Majewski and Cohan 1999; Eppley *et al.* 2007). Thus, the most important source of eDNA for homologous recombination would be the genomes of conspecific cells. For the sake of simplicity, the model assumed that genomes of conspecific cells in the same population were the only source of eDNA.

Prokaryotic cells can release their genomic DNA into the extracellular environment either actively by fratricide, suicide, or DNA secretion, or passively by natural death. However, the model assumed that genomic DNA was released only by natural cell death in order to consider the simplest and most unfavorable condition for the putative advantage of HGT (see the *Introduction* section).

To incorporate the influx of eDNA as the result of natural cell death, the model assumed that the per-generation probability of an individual with *i* mutations releasing its genome into the eDNA pool (*i.e.*, dying) was $\varepsilon_{i}=(1-f_{i}^{\prime })/\sum_{j=0}^{\infty }(1-f_{j}^{\prime })n_{j}$ where *n_j_* is the number of cells with *j* mutations, and $f_{j}^{\prime }$ is the fitness of cells with *j* mutations normalized by the fitness of the least-loaded class, *i.e.*, $f_{j}^{\prime }=f_{j}/f_{m_{\text{LLC}}}$ where *m*_LLC_ is the number of mutations in the least-loaded class (*m*_LLC_ is defined as the integer for which *n_m_* = 0 for *m* < *m*_LLC_ and $n_{m_{\text{LLC}}}>0$).

The aforementioned assumption on ε*_i_* was obtained from the following heuristic argument. Let us consider a continuous population dynamics described by *dn_i_*/*d_t_* = *bn_i_* − *d_i_n_i_*, where *b* and *d_i_* are the birth and death rates, respectively. The absolute fitness per unit time of a cell with *i* mutations can be expressed as $F_{i}=e^{b-d_{i}}$. The relative fitness is expressed as $f_{i}=F_{i}/e^{b-d_{0}}$ to be consistent with the aforementioned definition of *f*_i_ (so that *f*_0_ = 1). The fraction (probability) of cells dying per unit time is $1-e^{-d_{i}}$, which can be expressed as 1 − (1 − *D*_0_)*f*_i_, where $D_{0}=1-e^{-d_{0}}$ (*D*_0_ is the probability that an individual of the least-loaded class dies). Thus, the normalized contribution to the eDNA pool by a cell with *i* mutations is $(1-(1-D_{0})f_{i})/\sum_{j}(1-(1-D_{0})f_{j})n_{j}$. Setting *D*_0_ to zero maximizes the number of mutations in the genomes of dead cells (see Supporting Information, File S1), leading to the expression $\varepsilon_{i}^{\prime }=(1-f_{i})/\sum_{j=0}^{\infty }(1-f_{j})n_{j}$. This expression is used in the seminal work of Redfield (Redfield 1988), which considers an infinite population model. In a finite population model, however, the number of mutations in the least-loaded class can increase over time owing to Muller’s ratchet. To keep constant the average number of mutations in the eDNA pool relative to the number of mutations in the least-loaded class (so that the dynamics is stationary over time), the fitness in $\varepsilon_{i}^{\prime }$ was normalized by the fitness of the least leaded class $f_{m_{\text{LLC}}}$ to obtain the expression for ε*_i_* shown previously. Note that assuming ε*_i_* is more unfavorable to the hypothesized advantage of HGT than assuming $\varepsilon_{i}^{\prime }$ because $\varepsilon_{i}^{\prime }$ is nearly independent of *i* when $m_{\text{LLC}}\gg 1$.

In addition, the model assumed spontaneous decay of eDNA at rate $d_{\text{eDNA}}$ per cell generation. The eDNA decay rate determines the turnover of the eDNA pool, *i.e.*, how synchronous the genetic content of the eDNA pool is relative to that of the population (*e.g.*, when $d_{\text{eDNA}}=1$, the entire eDNA pool is replaced with the new input every generation). Although the absolute concentration of eDNA is likely to influence the frequency of HGT (Levin and Cornejo 2009), in the model this influence was assumed to be absorbed by *r* so that the value of *d*_eDNA_ does not influence the frequency of HGT. To indicate this fact, *d*_eDNA_ will be henceforth referred to as the rate of eDNA turnover.

With the aforementioned assumptions, the dynamics of the eDNA pool was simulated as follows. The state of the eDNA pool was described by the vector $\left(e_{10},e_{11},\cdots e_{ij}\cdots \right)$ where *e*_ij_ is the copy number of allele *j* (*i.e.*, *j* mutations) from locus *i* present in the eDNA pool. In each generation, $e_{ij}$ reduced by *d*_eDNA_*e_ij_* (if *d*_eDNA_*e_ij_* > 1, it was normalized to the nearest integer; otherwise, the decrease of *e_ij_* was drawn from a binomial distribution with the number of trials *e_ij_* and success probability *d*_eDNA_). Then, *N* cells were randomly chosen with replacement from the population of the current generation with the probability ε*_i_*. All alleles in the genomes of the chosen cells were added to the eDNA pool, leading to the increase of *e_ij_*.

### Population structure

The simplest version of the model assumed that the population is genetically isolated from other populations. However, to investigate the effect of genetic exchange between semi-isolated populations, we also considered an extended model in which the population was subdivided into 16 subpopulations of an equal size. The subpopulations were arranged in a two-dimensional square grid (4 × 4 subpopulations) with toroidal boundaries. Each subpopulation exchanged individuals with the four nearest-neighbor subpopulations via random migration. The number of migrants between each pair of neighboring subpopulations was drawn from the binomial distribution with the success probability *D*_pop_ and *N*_s_/4 trials where *N*_s_ is the size of a subpopulation (*N*_s_ = *N*/16). Each subpopulation had its own pool of eDNA that exchanged alleles with the four nearest-neighbor pools via diffusion. The copy number of each allele diffusing out of a pool was defined as a fraction *D*_eDNA_/4 per direction per generation (if *D*_eDNA_*e_ij_*/4 > 1, it was normalized to the nearest integer; otherwise, the numbers of copies diffusing out were drawn from a multinomial distribution with the number of trials *e_ij_* and success probability *D*_eDNA_/4 per direction).

### Organization of the simulation

The dynamics of the model in one generation was simulated in four steps, in the following order:

1. The Wright-Fisher process. In this step, the population of the current generation died and was replaced by that of the next generation.
2. Production and decay of the eDNA pools.
3. Diffusion of both the microbial populations and eDNA.
4. Mutation and HGT.

## Results

### Characterization of Muller’s ratchet

With HGT disabled, the model is known to have the following family of stationary distributions $\bar{n}=({\bar{n}}_{0},{\bar{n}}_{1},\cdots )$ that are distinguished by the number of deleterious mutations in the least-loaded class $m_{\text{LLC}}$: ${\bar{n}}_{0}=0$, ⋯, ${\bar{n}}_{m_{\text{LLC}}-1}=0$, ${\bar{n}}_{m_{\text{LLC}}+k}=Ne^{-U/s}{\left(U/s\right)}^{k}/k!$ where *k* is a non-negative integer (Haigh 1978). A finite-size population cannot stay in any of these distributions indefinitely because of stochasticity. The absence of back mutations implies that *m*_LLC_ increases over time, hence the accumulation of deleterious mutations (Haigh 1978). In general, such accumulation can result from two different processes (Charlesworth *et al.* 1993): the fixation of deleterious mutations via genetic drift or the operation of Muller’s ratchet *per se*, *i.e.*, the extinction of the least-loaded class as the result of stochastic fluctuations. Muller’s ratchet can be reversed by recombination only if a mutation has not been fixed (unless eDNA turnover is very slow, as described later in this article). Thus, it is beneficial to know through which of these processes the accumulation of mutations occurs in the model. Thus, we first analyzed the model in the absence of HGT (*i.e.*, *r* = 0). The results of the analysis essentially confirmed the conclusion of Charlesworth and Charlesworth (1997) with additional data. Although these results in part reproduce the work of Charlesworth and Charlesworth (1997), we present them here because they serve as the baseline with which to compare the results presented in the later sections.

**Figure 1:**
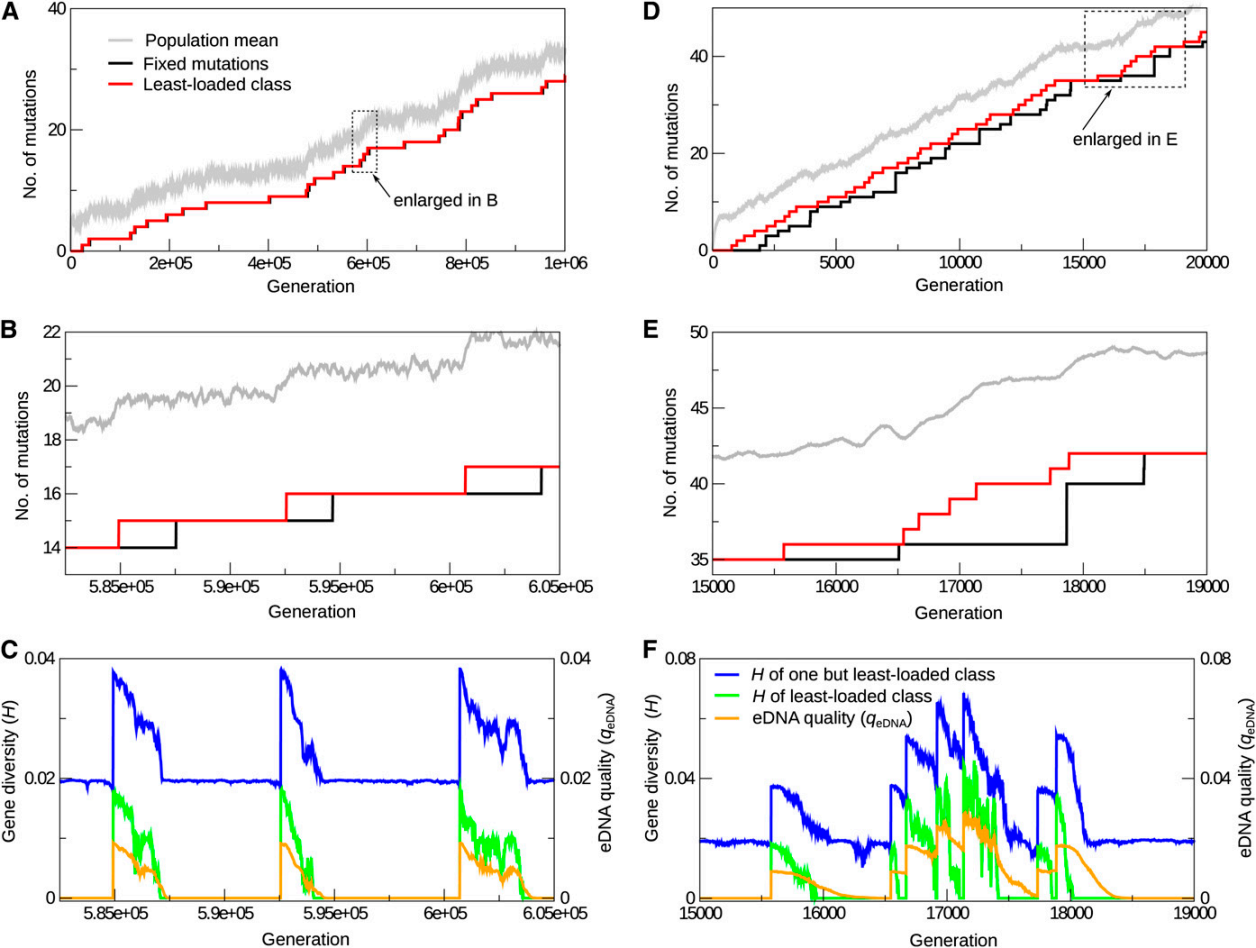
Operation of Muller’s ratchet in the model in the absence of HGT (r=0). (A, B, D, E) The number of deleterious mutations is plotted against time: the population average (gray); the number of mutations fixed in the population (black); and the number of mutations in the least-loaded class (red). (C and F) The average gene diversity of the least-loaded class $H_{m_{\text{LLC}}}$ (green), that of the one but the least-loaded class $H_{m_{\text{LLC}}+1}$ (blue), and the eDNA quality $q_{\text{eDNA}}$ (orange) are plotted against time (see the main text for how these quantities are defined). Parameters were as follows: *s* = 0.01, *N* = 10^5^, *d*_eDNA_ = 1, *l* = 100. In the slow ratchet regime, $s{\bar{n}}_{m_{\text{LLC}}}=10$, $U=4.6052\times 10^{-2}$ (A−C); in the fast ratchet regime, $s{\bar{n}}_{m_{\text{LLC}}}=1$, $U=6.9078\times 10^{-2}$ (D−F).

The simulations show that the tempo and mode of mutation accumulation in the model depend on whether the value of $s{\bar{n}}_{m_{\text{LLC}}}$ ($=sNe^{-U/s}$) is much greater than unity or not, as expected from previous work (Rouzine *et al.* 2008; Neher and Shraiman 2012). For $s{\bar{n}}_{m_{\text{LLC}}}=10$ (slow accumulation regime), *m*_LLC_ and the number of mutations fixed in the population (*m*_fix_) increase over time in a very similar fashion (Figure 1A), so that the number of segregating mutations in the least-loaded class (*m*_LLC_ − *m*_fix_) is at most one. Each increase in *m*_LLC_ is followed by an increase in *m*_fix_ with a delay of approximately a few thousand generations before the next increase occurs to *m*_LLC_ (Figure 1B). Thus, the extinction of the least-loaded class (*i.e.*, a click of Muller’s ratchet) always precedes the fixation of a mutation (Charlesworth and Charlesworth 1997).

**Figure 2:**
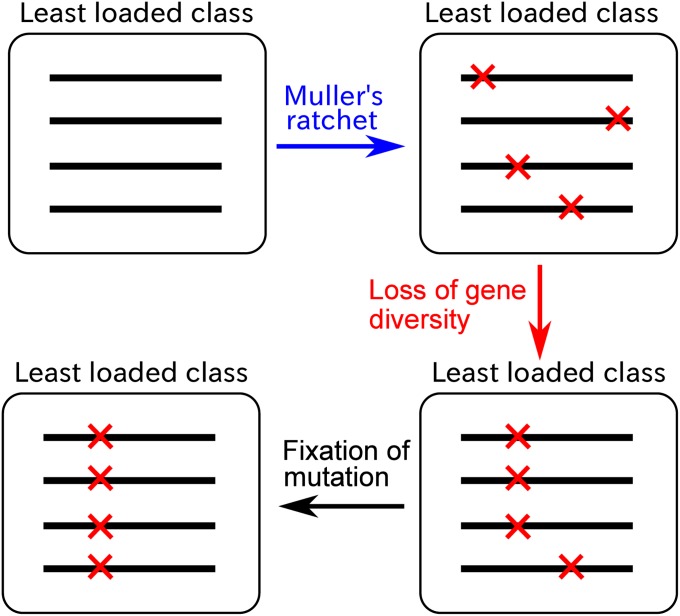
Schematic diagram depicting accumulation of mutations in the model. Horizontal bars indicate the genomes of the least-loaded class (genomes of the other classes in the population are not shown). Crosses on the bars indicate deleterious mutations.

The dynamics of mutation accumulation was further analyzed by measuring the average gene diversity (“heterozygosity”) of the least-loaded class (denoted $H_{m_{\text{LLC}}}$) and that of one but the least-loaded class ($H_{m_{\text{LLC}}+1}$; Nei 1987). $H_{m_{\text{LLC}}}$is defined as $H_{m_{\text{LLC}}}=\frac{1}{l}\sum_{i=1}^{l}\left(1-\sum_{j=0}^{\infty }x_{ij}^{2}\right)$ where $x_{ij}$ is the frequency of allele *j* in locus *i* in the least-loaded class (Nei 1987). $H_{m_{\text{LLC}}+1}$ is defined in a similar manner. The results show that $H_{m_{\text{LLC}}}$ and $H_{m_{\text{LLC}}+1}$ display the following dynamics. When Muller’s ratchet clicks (*i.e.*, when the value of *m*_LLC_ increases), one but the least-loaded class becomes the new least-loaded class by definition, so that the value of $H_{m_{\text{LLC}}}$ jumps to the value of $H_{m_{\text{LLC}}+1}$ immediately prior to the click of the ratchet (Figure 1C). This value of $H_{m_{\text{LLC}}+1}$ is always nearly 0.02, indicating that, immediately prior to a click of Muller’s ratchet, one but the least-loaded class consists of *l* genotypes (one mutation in one of the *l* loci), whose frequencies roughly equal to each other ($H_{m_{\text{LLC}}+1}=1-{\left(1/l\right)}^{2}-{\left(1-1/l\right)}^{2}=0.0198$ for l=100). Thus, immediately after the ratchet clicks, the new least-loaded class consists of *l* genotypes. Because these genotypes have an equal fitness value, the gene diversity $H_{m_{\text{LLC}}}$ decreases to zero over time owing to genetic drift (Figure 1C). Almost simultaneously with $H_{m_{\text{LLC}}}$ hitting zero, $H_{m_{\text{LLC}}+1}$ reaches a steady state value of approximately 0.02 mentioned previously (Figure 1C), and the value of *m*_fix_ increases, indicating the fixation of a deleterious mutation (Figure 1B). In summary, the accumulation of mutations occurs as a cycle of three distinct steps (Figure 2):

1. Extinction of the least-loaded class due to stochasticity (*i.e.*, Muller’s ratchet);
2. Gradual decline of the genetic diversity in the new least-loaded class due to genetic drift;
3. Eventual fixation of a deleterious mutation in the entire population.

For $s{\bar{n}}_{m_{\text{LLC}}}=1$, mutations accumulate more rapidly than when $s{\bar{n}}_{m_{\text{LLC}}}=10$ (Figure 1D). The value of *m*_LLC_ is incremented multiple times before the value of *m*_fix_ reaches the value of *m*_LLC_ (Figure 1E). This result indicates that, unlike the case of $s{\bar{n}}_{m_{\text{LLC}}}=10$, cycles of mutation accumulation can overlap with each other in time: a new cycle can begin with a click of Muller’s ratchet (*i.e.*, step 1 in the previous paragraph) before the previous cycle undergoes the fixation of a mutation (*i.e.*, step 3). Moreover, the dynamics of $H_{m_{\text{LLC}}}$ and $H_{m_{LLC}+1}$ are no more synchronized, in that the decrease of $H_{m_{LLC}+1}$ after a click of Muller’s ratchet is delayed with respect to the decrease of $H_{m_{\text{LLC}}}$ (cf. Neher and Shraiman 2012). Likewise, the increase of *m*_fix_ also is delayed with respect to the decrease of $H_{m_{LLC}+1}$. These results indicate that the decline of gene diversity after a click of Muller’s ratchet (*i.e.*, step 2) is a sequential process, in that gene diversity is consecutively lost in different genotype classes distinguished by the number of mutations (*i.e.*, first, $H_{m_{LLC}}$ decreases to zero, and then $H_{m_{LLC}+1}$ decreases to a steady-state level, and so on). Taken together, the results indicate that the three-step picture of mutation accumulation described in the previous paragraph remains essentially valid in the rapid ratchet regime ($s{\bar{n}}_{m_{\text{LLC}}}=1$) as well.

Therefore, Muller’s ratchet is the dominant process leading to the accumulation of mutations in the model with HGT disabled (rather than the fixation by drift of mutations; Charlesworth and Charlesworth 1997). However, a click of Muller’s ratchet eventually results in the fixation of a mutation, so that the number of fixed mutations (*m*_fix_) far exceeds the number of segregating mutations in the least-loaded class (*m*_LLC_ − *m*_fix_). This situation is in stark contrast with the result with a diploid, sexual model that assumes no recombination and weak dominance, which shows that the number of fixed mutations is much smaller than the number of segregating mutations (Charlesworth *et al.* 1993). The most probable cause of this discrepancy is the prevention of mutation fixation in diploid populations due to homozygote disadvantage, which is absent in our haploid model. The resulting paucity of segregating mutations in the haploid model seems to work against the putative advantage of HGT; later we show how this limitation might be overcome by population subdivision.

### Effect of HGT on Muller’s ratchet

We next examined the effect of HGT on Muller’s ratchet. For simplicity, a rapid turnover of eDNA was assumed by setting *d*_eDNA_ = 1 (the effect of slow eDNA turnover is presented in the next section). In this case, assuming a very large population, the average number of mutations in the eDNA pool per *l* loci (*i.e.*, per genome) can be approximated by *U*/*s* + 1, where *U*/*s* is the average number of mutations in the population at the steady state with *m*_LLC_ = 0 (Redfield *et al.* 1997; File S1). Because the mean number of mutations in the eDNA pool is greater than that in the population, HGT on average introduces more deleterious mutations into the population than it removes and so decreases the steady-state population size of the least-loaded class.

**Figure 3:**
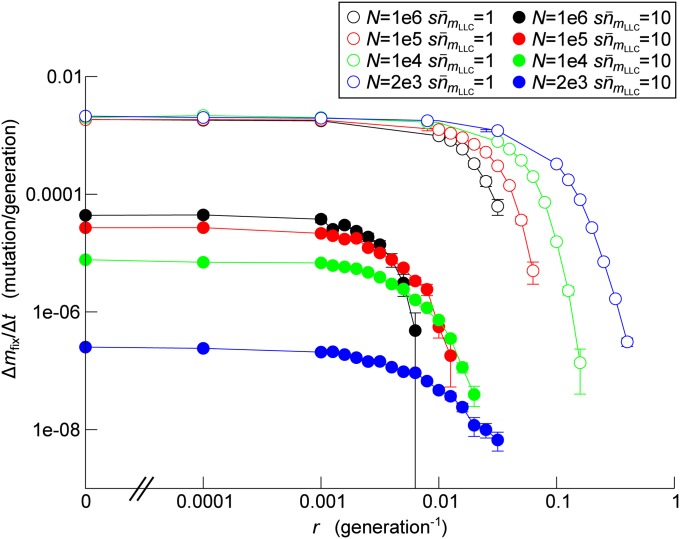
The effect of HGT on the operation of Muller’s ratchet. The rate of mutation accumulation Δ*m*_fix_/Δ*t* is plotted as a function of the rate of HGT *r*. The accumulation rate was estimated as *m*_fix_/*t*, where *m*_fix_ is the number of fixed mutations at the end of a simulation and *t* is the duration of a simulation (*e.g.*, *t* >10^6^ for *N* = 10^6^ and *t* >10^9^ for *N* = 2 × 10^3^). The error bars indicate standard error of means estimated as $\sqrt{m_{\text{fix}}/t^{2}}$. The parameters were as follows: *s* = 0.01, *d*_eDNA_ = 1, *l* = 100. In the slow ratchet regime, $s{\bar{n}}_{m_{\text{LLC}}}=10$ (filled circles): *N* = 10^6^ and $U=6.9078\times 10^{-2}$ (black), *N* = 10^5^ and $U=4.6052\times 10^{-2}$ (red), *N* = 10^4^ and $U=2.3026\times 10^{-2}$ (green), *N* = 2 × 10^3^ $U=6.9315\times 10^{-3}$ (blue). In the fast ratchet regime, $s{\bar{n}}_{m_{\text{LLC}}}=1$ (open circles): *N* = 10^6^ and $U=9.2103\times 10^{-2}$ (black), *N* = 10^5^ and $U=6.9078\times 10^{-2}$ (red), *N* = 10^4^ and $U=3.6052\times 10^{-2}$ (green), $N=2\times 10^{3}$ and $U=2.9957\times 10^{-2}$ (blue).

To assess the net effect of HGT on Muller’s ratchet, we measured the rate of mutation accumulation Δ*m*_fix_ /Δ*t* as a function of *r* (HGT rate) for different combinations of *N* and $s{\bar{n}}_{m_{\text{LLC}}}$ values. The results show that HGT prevents the accumulation of mutations (*i.e.*, Δ*m*_fix_ /Δ*t* = 0) if its frequency is sufficiently high (Figure 3). Moreover, the frequency of HGT required to stop Muller’s ratchet decreases with the increase of *N*, indicating that the preventive effect of HGT increases with *N*. This trend is seen even in the slow ratchet regime ($s{\bar{n}}_{m_{\text{LLC}}}=10$), in which mutation accumulation accelerates with the increase of *N* in the absence of HGT (Figure 3) (note that because $s{\bar{n}}_{m_{\text{LLC}}}$ and *s* are fixed, *U* was set to a greater value for a greater value of *N*; thus, mutation accumulation can accelerate with the increase of *N*).

To understand why HGT can prevent Muller’s ratchet despite its average deleterious effect due to the high mutation load of eDNA that comes from dead cells, we considered the following simple Wright-Fisher model. The model assumed two populations: the population of the least-loaded class *n*_0_ and that of the other classes *n*_1_ (the model is thus equivalent to one-locus, two-allele, haploid model). The total population size N = *n*_0_ + *n*_1_ was constant. The value of *n*_0_ at time *t* + 1 was drawn from a binomial distribution with *N* trials and success probability *p*′ defined as follows:

$$p^{\prime }=(1-U)(1-r_{01})p/\bar{w}+(1-s^{\prime })r_{10}q/\bar{w}$$

where *p* = *n*_0_/*N* and $q=n_{1}/N$ (=1−p) at time *t*, *s*′ is the fitness effect of mutation, *U* is the mutation rate from *n*_0_ to *n*_1_ (back mutations ignored), $\bar{w}$ is the average fitness, and *r*_01_ and *r*_10_ are the rates of HGT. For simplicity, HGT was assumed to convert *n*_0_ into *n*_1_ at rate *r*_01_ and, conversely, *n*_1_ into *n*_0_ at rate *r*_10_. The effect of eDNA was implicitly incorporated into the model by the choice of parameters *r*_01_ and *r*_10_ as follows. If *r*_01_ and *r*_10_ satisfy the following condition, HGT decreases the steady-state level of *n*_0_ compared with the steady-state level reached in the absence of HGT (*i.e.*, *r*_01_ = *r*_10_ = 0):

$$r_{10}(1-s^{\prime })U^{\prime }/s^{\prime }<r_{01}(1-U^{\prime })(1-U^{\prime }/s^{\prime })$$

(the left-hand side of this inequality is the per-generation conversion of *n*_1_ into *n*_0_, whereas the right-hand side is that of *n*_0_ into *n*_1_, when *n*_0_ and *n*_1_ are set to the steady-state level achieved in the absence of HGT).

**Figure 4:**
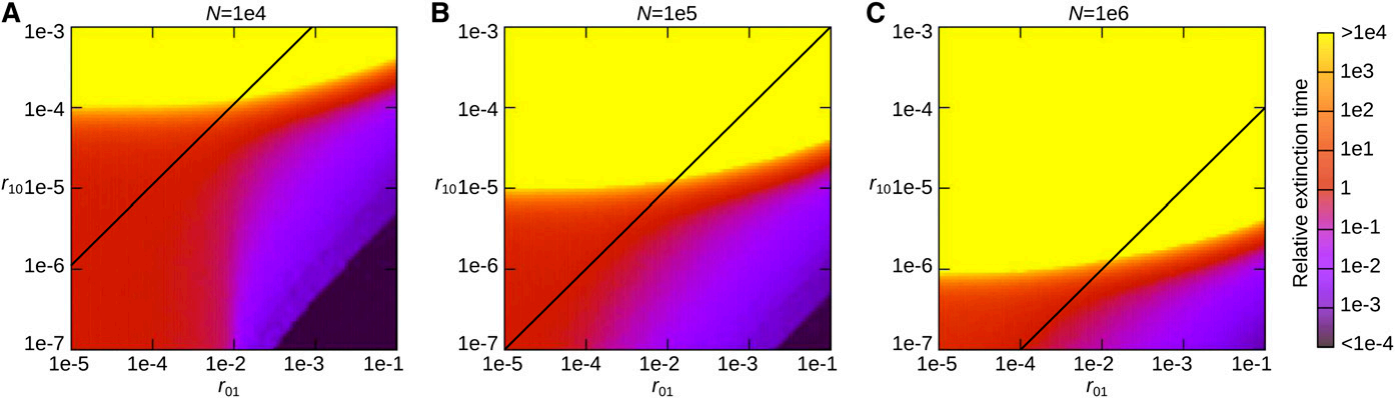
The mean time to extinction of the least-loaded class in the simplified Fisher-Wright model. Below the diagonal lines, HGT decreases the steady-state population size of the least-loaded class (*i.e.*, the condition $r_{10}(1-s^{\prime })U^{\prime }/s^{\prime }<r_{01}(1-U^{\prime })(1-U^{\prime }/s^{\prime })$ is fulfilled). The mean time to extinction is normalized by that obtained in the absence of HGT (*i.e.*, $r_{01}=r_{10}=0$). The parameters were as follows: $s^{\prime }=0.01$, and $s^{\prime }{\bar{n}}_{0}=10$ where ${\bar{n}}_{0}=N(1-U^{\prime }/s^{\prime })$. *N* = 10^4^ and $U^{\prime }=9\times 10^{-3}$ (A); *N* = 10^5^ and $U^{\prime }=9.9\times 10^{-3}$ (B); *N* = 10^6^ and $U^{\prime }=9.99\times 10^{-3}$ (C). Each data point was obtained as an average of 100 simulation runs.

With this simplified model, we asked whether HGT facilitated or antagonized the survival of *n*_0_. The mean time to extinction of *n*_0_, which is a proxy for Δ*m*_fix_/Δ*t* in the full model, was measured as a function of *r*_01_ and *r*_10_ for different combinations of *N* and *U*′ values. The results show that HGT increases the mean time to extinction of *n*_0_ whether or not HGT decreases the steady state level of *n*_0_, as long as the per-generation production of the least-loaded class by HGT ($r_{10}{\bar{n}}_{1}$) is on the order of unity or greater (Figure 4; note that ${\bar{n}}_{1}\approx N$ for the parameters used in the simulations). This result makes intuitive sense: the least-loaded class cannot go extinct if it is continually produced by HGT in every generation; and as long as such production is guaranteed, the steady-state population size of *n*_0_ is immaterial to the extinction of *n*_0_. To check whether this explanation also applies to the full model, we investigated the consistency between the two models from a few different angles as described below.

First, the simplified model indicates that the required frequency of HGT to prevent Muller’s ratchet decreases as *N* increases because increasing *N* increases the production rate of the least-loaded class (with ${\bar{n}}_{0}$ kept constant). The same result is produced by the full model as already described previously.

**Figure 5:**
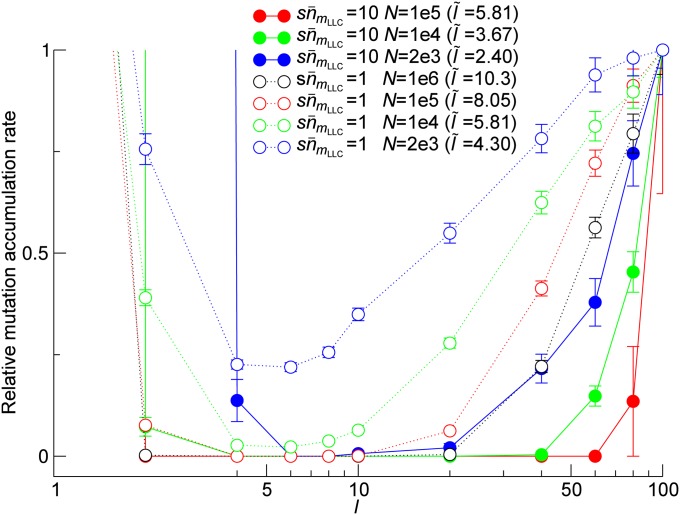
The normalized rate of mutation accumulation as a function of the number of loci *l*. The reference rate is set to the rate obtained for *l* = 100. The value of *l* that maximizes the probability *P*_10_ (that HGT converts one but the least-loaded class to the least-loaded class) is indicated as $\tilde{l}$ in the graph. The parameters and color coding are the same as in Figure 3 (except for *l*).

Next, Figure 4 shows that whether HGT increases the time to extinction depends on the value of *r*_10_ much more strongly than on the value of *r*_01_ (as seen from the fact that the boundary between yellow and red regions is nearly horizontal). We thus hypothesized that, in the full model, HGT prevents Muller’s ratchet most effectively when the probability of HGT producing the least-loaded class is maximized. This probability can be approximated by the probability *P*_10_ that an HGT event transforms one but the least-loaded class ($n_{m_{\text{LLC}}-1}$) into the least-loaded class ($n_{m_{\text{LLC}}}$), because the contributions from other mutant classes likely involve more than one HGT event. *P*_10_ can be calculated as $l^{-1}(1-l^{-1})e^{-l^{-1}U/s}$ under the assumption that *N* is very large (File S1). *P*_10_ takes the maximum value when $l^{-1}={\tilde{l}}^{-1}\equiv s/U+1/2-\sqrt{{\left(s/U\right)}^{2}+1/4}$ (${\tilde{l}}^{-1}\approx s/U+{\left(s/U\right)}^{2}$ when s/U≪1; File S1). The non-monotonic dependency of *P*_10_ on *l* can be intuitively understood as follows: decreasing *l* can decrease *P*_10_ because it increases the probability that an allele randomly drawn from the eDNA pool contains a mutation that is not carried by the least-loaded class (if *l* = 1, this probability is one). However, increasing *l* also can decrease *P*_10_ because it decreases the probability that HGT occurs to the very locus in which one but the least-loaded class has the mutation not carried by the least-loaded class. Therefore, *P*_10_ takes the maximum value at an intermediate value of *l*. By contrast, the probability *P*_01_ that HGT introduces one or more mutations in the least-loaded class is $1-(1-l^{-1})e^{-l^{-1}U/s}$ (File S1), which is a monotonically decreasing function of *l* because increasing *l* decreases the probability that an allele randomly drawn from the eDNA pool contains mutations. Thus, the aforementioned hypothesis can be reformulated as follows: Δ*m*_fix_/Δ*t* reaches the minimum value when *l* assumes the value $\tilde{l}$. To examine this hypothesis, we measured Δ*m*_fix_/Δ*t* as a function of *l* for various combinations of *N* and $s{\bar{n}}_{0}$ (Figure 5). The results show that Δ*m*_fix_/Δ*t* takes the minimum value when the value of *l* is on the same order of magnitude as $\tilde{l}$ (around 10 rather than 100), in accord with the hypothesis.

Finally, the simplified model shows that $r_{10}{\bar{n}}_{1}\geq 1$ is sufficient for HGT to prevent the extinction of $n_{0}$. To check if a similar condition applies to the full model, we calculated the per-generation production of the least-loaded class by HGT occurring to one but the least-loaded class ($rP_{10}{\bar{n}}_{1}$). For $s{\bar{n}}_{m_{\text{LLC}}}=10$, $\Delta m_{\text{LLC}}/\Delta t$ sharply drops at r≈0.01 for *N* = 10^6^ and 10^5^ (Figure 3). The values of $rP_{10}{\bar{n}}_{m_{\text{LLC}}}$ in these cases are approximately 0.6 and 0.4 for *N* = 10^6^ and 10^5^, respectively, in qualitative agreement with the expectation that $rP_{10}{\bar{n}}_{m_{\text{LLC}}}$ be on the order of unity (note that $rP_{10}{\bar{n}}_{m_{\text{LLC}}}<1$ is expected because the contributions from the more-loaded classes are ignored and $n_{m_{\text{LLC}}}>0$ is a stronger condition than $\Delta m_{\text{fix}}/\Delta t=0$). For $s{\bar{n}}_{m_{\text{LLC}}}=1$, $\Delta m_{\text{LLC}}/\Delta t$ sharply drops at r≈0.1 for *N* = 10^6^ and 10^5^. The values of $rP_{10}{\bar{n}}_{m_{\text{LLC}}}$ in these cases are approximately 0.8 and 0.6 for *N* = 10^6^ or 10^5^, respectively, again in qualitative agreement with the expectation. (The results were also consistent for smaller values of *N*, at least in the orders of magnitude.)

Taken together, the consistency of the results indicates that the simplified model captures, at least qualitatively, the mechanism by which HGT prevents Muller’s ratchet in the full model. Namely, HGT can prevent Muller’s ratchet even if it decreases the steady-state population size of the least-loaded class because it can hinder the extinction of the least-loaded class by continually generating it from the more-loaded classes.

### Effect of the eDNA turnover rate on the prevention of Muller’s ratchet by HGT

We further investigated the effect of the eDNA turnover rate on the ability of HGT to prevent Muller’s ratchet. As described previously, the accumulation of mutations in the model is initiated by a click of Muller’s ratchet followed by the fixation of a mutation. Once a mutation is fixed (*i.e.*, once a good allele is lost), HGT cannot undo it because the only source of HGT in the model is eDNA derived from the members of the same population. However, slow turnover of eDNA can delay the loss of good alleles from the eDNA pool and thereby might help HGT prevent the accumulation of mutations.

**Figure 6:**
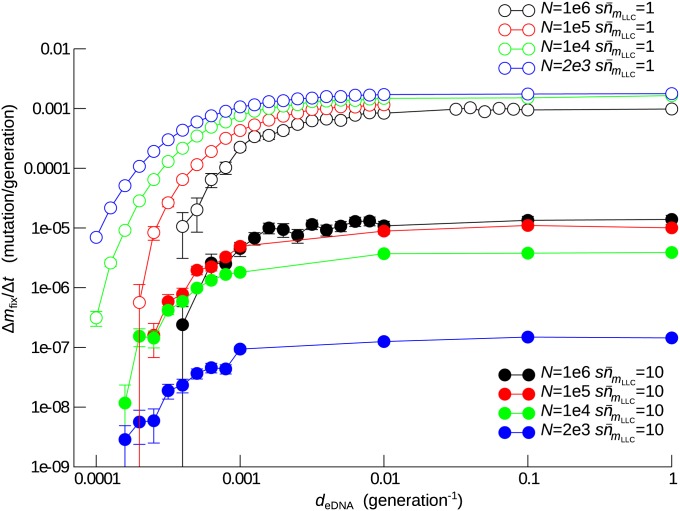
Effect of slow eDNA turnover on the prevention of Muller’s ratchet by HGT. The rate of mutation accumulation Δ*m*_fix_/Δ*t* is plotted as a function of the eDNA turnover rate *d*_eDNA_. The parameters and color coding are the same as in Figure 3 (except for *d*_eDNA_).

To examine this possibility, we measured $\Delta m_{\text{fix}}/\Delta t$ as a function of *d*_eDNA_ for various combinations of *N* and $s{\bar{n}}_{m_{\text{LLC}}}$ values (Figure 6). The results show that Δ*m*_fix_/Δ*t* decreases with the decrease of *d*_eDNA_. However, for this effect to be significant, eDNA turnover has to be slower than the population turnover (*i.e.*, generation time) by more than three orders of magnitude (*d*_eDNA_ < 10^−3^), a condition that appears to be unrealistic in natural environments (Nielsen *et al.* 2007; Pietramellara *et al.* 2009).

**Figure 7:**
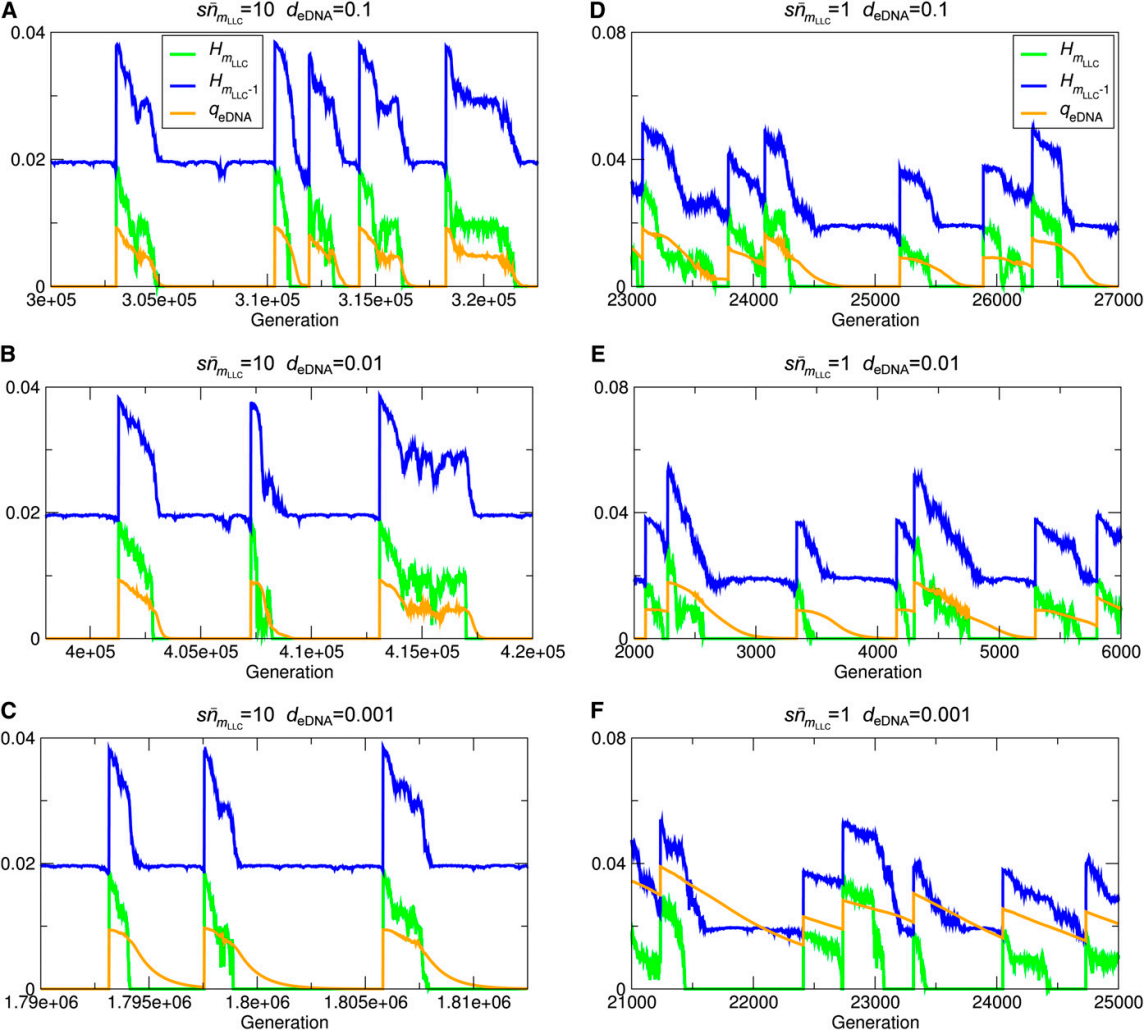
Effect of slow eDNA turnover on the quality of eDNA *q*_eDNA_ . The average gene diversity of the least-loaded class $H_{m_{\text{LLC}}}$ (green), that of the one but the least-loaded class $H_{m_{\text{LLC}}+1}$ (blue), and the quality of eDNA *q*_eDNA_ (orange) are plotted against time for various values of eDNA turnover rate *d*_eDNA_. The parameters were as follows: *l* = 100, *s* = 0.01, *d*_eDNA_ = 0.1 (A and C), 0.01 (B and E), and 0.001 (C and F). In the slow ratchet regime, $sNe^{-U/s}=10$, *N* = 10^5^ and $U=4.6052\times 10^{-2}$(A−C). In the fast ratchet regime, $sNe^{-U/s}=1$, *N* = 10^5^, and $U=6.9078\times 10^{-2}$ (D−F).

To understand why such a severe condition applied, we measured the “quality” of eDNA defined as $q_{\text{eDNA}}=\sum_{k=1}^{l}\sum_{j=0}^{\infty }\sum_{i=j+1}^{\infty }(i-j)x_{ki}y_{kj}$ where *x_ki_* is the fraction of the least-loaded class having allele *i* (*i.e.*, *i* mutations) in locus *k*, and $y_{kj}$ is the fraction of allele *j* from locus *k* in the eDNA pool. *q*_eDNA_ indicates the potential of the eDNA pool to remove deleterious mutations in the genomes of the least-loaded class (*e.g.*, *q*_eDNA_ is zero if the eDNA pool contains no allele that can remove mutations in the least-loaded class). *q*_eDNA_ was measured in the absence of HGT for various values of *d*_eDNA_. The results show that, in the slow ratchet regime ($s{\bar{n}}_{0}=10$), the dynamics of *q*_eDNA_ closely follows that of $H_{m_{LLC}}$ when eDNA turnover is not very slow (*d*_eDNA_ ≥ 10^−2^) (Figure 1C and Figure 7, A and B). When eDNA turnover is very slow (*d*_eDNA_ ≤ 10^−3^), the decay of *q*_eDNA_ lags behind the decline of $H_{m_{LLC}}$ (Figure 7C). This result indicates that eDNA turnover has to be slower than the decline of $H_{m_{LLC}}$ to delay the decay of *q*_eDNA_ significantly. Because the decrease of $H_{m_{LLC}}$is attributed to genetic drift as mentioned previously, its timescale is determined by the population size of the least-loaded class, which was approximately 1000 in the simulations for the slow ratchet regime ($s{\bar{n}}_{m_{\text{LLC}}}=10$ and ${\bar{n}}_{m_{\text{LLC}}}=1000$). Thus, *d*_eDNA_ must be <10^−3^ to cause any significant effect.

The aforementioned argument might imply that the effect of slow eDNA turnover could be more significant if the population size of the least-loaded class is small. However, this turns out not to be the case. In the fast ratchet regime ($s{\bar{n}}_{m_{\text{LLC}}}=1$ and ${\bar{n}}_{m_{\text{LLC}}}=100$), the dynamics of *q*_eDNA_ no more closely follows the dynamics of $H_{m_{LLC}}$, and it still takes more than 500 generations for *q*_eDNA_ to decrease to zero when eDNA turnover is rapid (Figure 7, D−F). Therefore, *d*_eDNA_ still has to be smaller than 1/500 to have any significant effect (File S1 contains a more detailed explanation of the results described in this and the previous paragraphs).

In summary, the fixation of mutations is an evolutionary process that is driven by selection and drift and thus occurs on relatively long timescales compared with the generation time of individuals, so that eDNA turnover has to be commensurately slow to have any appreciable effect.

### Effect of population subdivision on the prevention of Muller’s ratchet by HGT

Next, we considered population subdivision. On the one hand, population subdivision can accelerate accumulation of mutations because it enhances the effect of genetic drift as shown in previous studies (Higgins and Lynch 2001; Combadao *et al.* 2007). On the other hand, subdivision might help HGT prevent Muller’s ratchet as follows. Let us suppose that the isolation between subpopulations is so strong that the accumulation of mutations in different subpopulations is independent of each other in the absence of HGT. In this case, a mutation must be fixed in every subpopulation independently before being fixed in the entire population. This situation is likely to lead to an increased number of segregating mutations when the entire population is considered (rather than when each subpopulation is considered separately). The increased number of segregating mutations by itself does not affect accumulation of mutations if recombination requires direct contact between individuals as in many animals. HGT, however, does not require such direct contact. If subpopulations share a common eDNA pool because of the rapid transport of DNA molecules between their habitats, increasing the number of segregating mutations is expected to increase the number of mutations that are introduced or removed by HGT events. This should enhance the preventive effect of HGT on Muller’s ratchet because removal of mutations by HGT is more important than introduction of mutations by HGT according to the simplified Wright-Fisher model presented above. The key question is whether this potential advantage of population subdivision can outweigh the disadvantage of population subdivision associated with the enhanced genetic drift.

**Figure 8:**
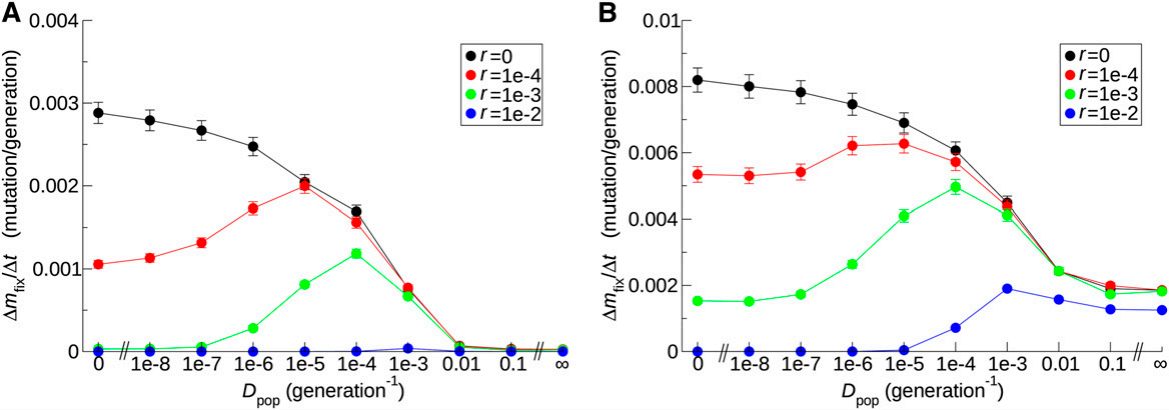
Effect of population subdivision on the prevention of Muller’s ratchet by HGT when eDNA diffusion is infinitely fast (*D*_eDNA_ = ∞). The rate of mutation accumulation $\Delta {\bar{m}}_{\text{fix}}/\Delta t$ is plotted as a function of population migration rate *D*_pop_ for various values of HGT rate *r*. The color coding is as follows: *r* = 0 (black), *r* = 10^−4^ (red), *r* = 10^−3^ (green), and *r* = 10^−2^ (blue). The parameters were as follows: in the slow ratchet regime, $sNe^{-U/s}=10$, *N* = 10^5^, and $U=4.6052\times 10^{-2}$ (A) ; in the fast ratchet regime, $sNe^{-U/s}=1$, *N* = 10^5^ , and $U=6.9078\times 10^{-2}$ (B). The number of subpopulations was 4×4 with toroidal boundaries (see *Materials and Methods*).

To address the aforementioned question, we measured $\Delta {\bar{m}}_{\text{fix}}/\Delta t$ as a function of population migration rate (*D*_pop_) in a model incorporating population subdivision (*Materials and Methods*). ${\bar{m}}_{\text{fix}}$ was defined as the average of *m*_fix_ measured within each subpopulation. For simplicity, all subpopulations were assumed to share a common eDNA pool (*i.e.*, *D*_eDNA_ = ∞; we will consider finite *D*_eDNA_ later). The results show that, in the absence of HGT (*r* = 0), the system becomes more prone to Muller’s ratchet (*i.e.*, $\Delta {\bar{m}}_{\text{fix}}/\Delta t$ increases) as *D*_pop_ decreases (Figure 8, A and B). This outcome is expected, given the disadvantage of population subdivision due to enhanced genetic drift. By contrast, in the presence of HGT (*r* ≥ 10^−4^), the dependency of $\Delta {\bar{m}}_{\text{fix}}/\Delta t$ on *D*_pop_ is non-monotonic: the system initially becomes more prone to Muller’s ratchet as *D*_pop_ decreases, but becomes less so as *D*_pop_ decreases further, with the position of the maximum $\Delta {\bar{m}}_{\text{fix}}/\Delta t$ along the *D*_pop_ axis shifting as a function of *r* (Figure 8, A and B). The comparison against the curve for *r* = 0 shows that the reduction in $\Delta {\bar{m}}_{\text{fix}}/\Delta t$ caused by HGT increases as *D*_pop_ decreases, indicating that population subdivision increases the effect of HGT. At a high HGT rate (*r* = 0.01), in the fast ratchet regime ($s{\bar{n}}_{m_{\text{LLC}}}=1$), the system is more resistant to Muller’s ratchet at lower values of *D*_pop_ (≤10^−5^) than at greater values of *D*_pop_ (≥10^−1^) for which the system can be considered well-mixed (Figure 8B). In the slow ratchet regime ($s{\bar{n}}_{m_{\text{LLC}}}=10$), however, this situation cannot be observed because $\Delta {\bar{m}}_{\text{fix}}/\Delta t$ falls to zero and so shows no difference between low and high values of *D*_pop_ within the accuracy of the simulations (Figure 8A). In addition to the aforementioned results, $\Delta {\bar{m}}_{\text{fix}}/\Delta t$ was measured as a function of *D*_pop_ for other combinations of *N* and $s{\bar{n}}_{m_{\text{LLC}}}$ values; the results were qualitatively the same as described previously (File S1). Taken together, these results indicate that the advantage of population subdivision can outweigh the associated disadvantage, provided that the frequency of HGT is sufficiently high, and subpopulations share a common eDNA pool.

**Figure 9:**
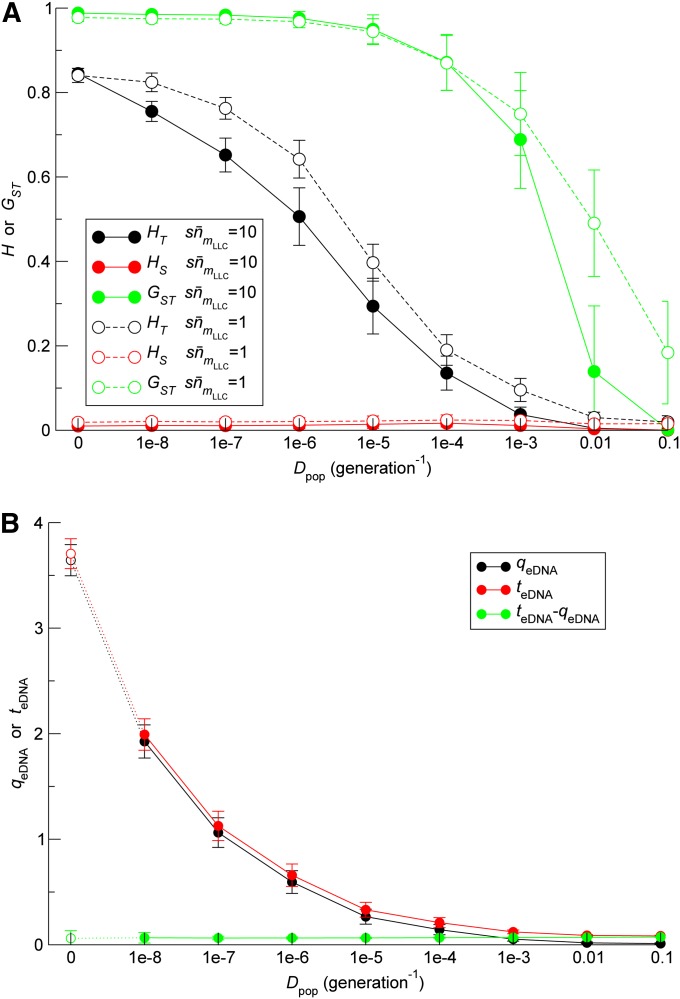
Effect of population subdivision on the genetic structure of populations and on the contents of eDNA pools. (A) The average gene diversity within subpopulations (*H_S_*) and in the entire population (*H_T_*), and the coefficient of gene differentiation G_ST_ = (*H_T_* − *H_S_*)/*H_T_* are plotted as a function of *D*_pop_ in the absence of HGT (*r* = 0). The plotted values were obtained as the time average after or nearly after the values reached saturation (all time averages were taken from the last $2\times 10^{4}$ generations of simulations). The error bars denote standard deviation. The parameters were the same as in Figure 8A (filled circles) and Figure 8B (open circles) except that *r* = 0. (B) The quality *q*_eDNA_ and toxicity *t*_eDNA_ of eDNA are plotted as a function of *D*_pop_ in the absence of HGT (*r* = 0). The values of *q*_eDNA_ and *t*_eDNA_ were obtained as the time average after the values reached saturation except for *D*_pop_ = 0, for which the values indicate lower bounds (all time averages were taken from the last 2 × 10^5^ generations of simulations). The error bars denote standard deviation. The parameters were the same as in Figure 8B except that *r* = 0.

To elucidate the mechanism by which population subdivision helps HGT prevent Muller’s ratchet, we first sought to test the expectation that population subdivision increases the number of segregating mutations at the level of the entire population. To this end, we measured the average gene diversity of the least-loaded class within and between subpopulations (Nei 1987). The average gene diversity within one subpopulation is defined as $H_{Sy}=(1/l)\sum_{k=1}^{l}(1-\sum_{i=0}^{\infty }x_{yki}^{2})$ where *x_yki_* is the frequency of allele *i* in locus *k* in the least-loaded class of subpopulation *y* (the least-loaded class was defined separately for each subpopulation). The average gene diversity within subpopulations *H_S_* is defined as the average *H_Sy_* over all subpopulations (weighted by the population size of the least-loaded class in each subpopulation). The average gene diversity in the entire population *H_T_* is defined similarly with *x_yk_*_i_ replaced by the frequency of an allele in the least-loaded classes from all subpopulations combined together. The average gene diversity between subpopulations was defined as *H_T_* − *H_S_* (Nei 1987). *H_T_* and *H_S_* were measured in the absence of HGT. Simulations show that the values of *H_T_* and *H_S_* initially increase and then saturate over time (results not shown). The time averages of *H_T_* and *H_S_* after or near saturation were obtained as a function of *D*_pop_ (Figure 9A). The result shows that *H_T_* increases as *D*_pop_ decreases, whereas *H_S_* remains close to zero for the entire range of *D*_pop_. This result indicates that population subdivision increases the number of loci that are polymorphic between subpopulations, whereas within subpopulations almost all loci remain monomorphic. Therefore, population subdivision increases the number of segregating mutations at the level of the entire population as expected.

The increase in the number of segregating mutations is expected to drive an increase in the number of mutations that are introduced or removed by HGT events. To test this expectation, we measured the quality of the eDNA pool defined above (*q*_eDNA_) and the “toxicity” of the eDNA pool defined as $t_{\text{eDNA}}=\sum_{k=1}^{l}\sum_{j=1}^{\infty }\sum_{i=0}^{j-1}(j-i)x_{ki}y_{kj}$ (*q*_eDNA_ and *t*_eDNA_ were calculated for each subpopulation and averaged over subpopulations using the population sizes of the least-loaded classes as weights). *t*_eDNA_ indicates the potential of the eDNA pool to introduce deleterious mutations in the genomes of the least-loaded class. Note that the difference *t*_eDNA_ − *q*_eDNA_ is equal to the average number of mutations introduced (or removed if the value is negative) by one HGT event occurring to an individual of the least-loaded classes. Thus, − *q*_eDNA_ can be interpreted as the contribution to the average number of mutations introduced by HGT by those HGT events that decrease the number of mutations, whereas *t*_eDNA_ as the contribution by those events that increase the number of mutations. Simulations show that the values of *q*_eDNA_ and *t*_eDNA_ increase and then saturate over time except for *D*_pop_ = 0 (result not shown; for *D*_pop_ = 0, saturation is likely to be reached, but probably requires an exceedingly long time). The time averages of *q*_eDNA_ and *t*_eDNA_ after saturation were obtained as a function of *D*_pop_ in the absence of HGT (Figure 9B; for *D*_pop_ = 0, the values reached toward the end of the simulation are shown, thus indicating a lower bound). The result shows that population subdivision increases the number of mutations removed by an HGT event (*q*_eDNA_) as well as the number of mutations introduced by an HGT event (*t*_eDNA_), thus confirming the expectation (Figure 9B). Moreover, it leaves nearly constant the average (*i.e.*, net) number of mutations introduced by an HGT event (*t*_eDNA_ − *q*_eDNA_). In other words, population subdivision increases the conversion of the least-loaded class by HGT in the direction of both increasing and decreasing the number of mutations without changing the magnitude and direction of the net conversion. According to the simplified Wright-Fisher model described before, removal of mutations by HGT (*r*_10_) has a greater influence on the extinction of the least-loaded class than introduction of mutations by HGT (*r*_01_). Although *r*_10_ and *r*_01_ are concerned with HGT occurring in different genotypes, they are analogous to *q*_eDNA_ and *t*_eDNA_, respectively. This analogy points to the advantage of increasing *q*_eDNA_ and *t*_eDNA_ with *t*_eDNA_ − *q*_eDNA_ kept constant to prevent Muller’s ratchet.

**Figure 10:**
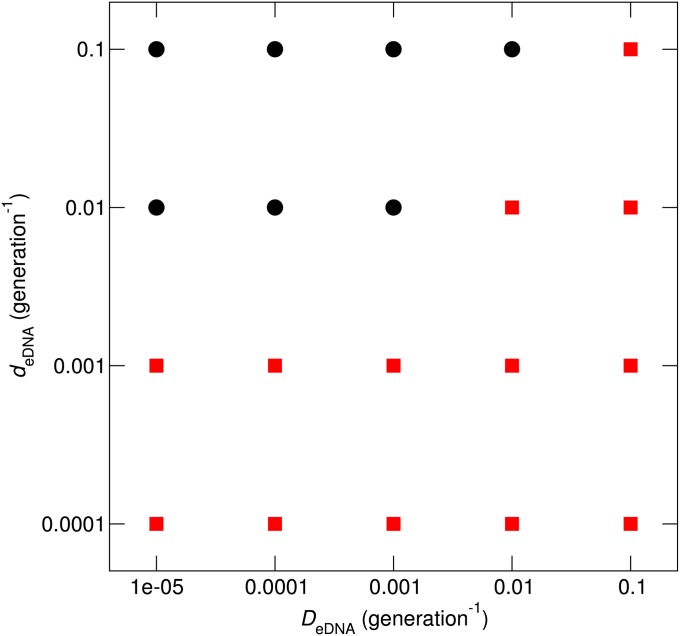
Effect of population subdivision on the prevention of Muller’s ratchet by HGT when the eDNA diffusion rate is finite. Red squares indicate the combinations of *d*_eDNA_ and *D*_eDNA_ values for which the rate of mutation accumulation $\Delta {\bar{m}}_{\text{fix}}/\Delta t$ is lower when a population is subdivided (*D*_pop_ = 10^−5^) than when a population is well-mixed (*D*_pop_ = 10^−1^). Black circles indicate the parameter region where the opposite was the case. The parameters (other than *D*_eDNA_ and *2*_eDNA_) were the same as in Figure 8B with r=0.01.

Whether population subdivision is advantageous or disadvantageous depends on the combination of parameters. To examine what appears to be the most important aspect of this parameter dependence, we next considered the finite diffusion of eDNA. To this end, the value of $\Delta {\bar{m}}_{\text{LLC}}/\Delta t$ was compared between *D*_pop_ = 10^−5^ (subdivided population) and *D*_pop_ = 10^−1^ (well-mixed population) for various combinations of *d*_eDNA_ and *D*_eDNA_ values. As expected, the results show that $\Delta {\bar{m}}_{\text{LLC}}/\Delta t$ is smaller for *D*_pop_ = 10^−5^ than for *D*_pop_ = 10^−1^ when *d*_eDNA_ is sufficiently small or *D*_eDNA_ is sufficiently large (Figure 10). The values of *D*_eDNA_ and *d*_eDNA_ demarcating the region of the parameter space for which population subdivision is advantageous do not appear unrealistic, although it is difficult to judge whether the conditions represented by this region are achievable in natural environments (see also *Discussion*).

## Discussion

The results presented here suggest that HGT can prevent the operation of Muller’s ratchet in prokaryotic populations, even if on average HGT introduces more deleterious mutations than it removes. The avoidance of Muller’s ratchet via transformation and recombination with eDNA might appear somewhat paradoxical because on average eDNA is expected to carry more deleterious mutations than the DNA in live prokaryotic cells. Indeed, it has been long recognized that “sex with dead cells” is a dubious proposition (Redfield 1988, 2001; Redfield *et al.* 1997). However, the modeling results indicate that, even though transformation via eDNA is expected to increase the mean mutation load and hence decrease the average fitness of a population, it nevertheless can stop Muller’s ratchet. This appears to be the case because HGT provides for the chance to eliminate deleterious mutations, leading to the continual restoration of the least-loaded class. In other words, HGT prolongs the persistence of the least-loaded class in the population in the face of stochastic fluctuations, hence the prevention of Muller’s ratchet.

The efficacy of HGT in the prevention of Muller’s ratchet depends on the stability of eDNA, its diffusion rate, and population subdivision. The contribution of eDNA stability (*i.e.*, slow turnover) is intuitively clear because it delays elimination of high-fitness alleles from the population and so increases the chance that these alleles offset Muller’s ratchet via HGT. However, the simulations show that, for this effect to be substantial, the characteristic lifetime of eDNA should exceed the generation time by orders of magnitude (Figure 6), suggesting that eDNA stability alone is not a key factor in the evolution of microbial populations. If, however, a population is subdivided, enhanced eDNA stability facilitates the transport of eDNA across subpopulations and thus contributes to the ability of HGT to prevent Muller’s ratchet. Under these conditions, for the effect to be significant, the characteristic lifetime of eDNA does not have to be exceedingly long, depending on the diffusion rate of eDNA (Figure 10).

If the transport of eDNA is sufficiently fast, HGT can prevent Muller’s ratchet more efficiently in a subdivided population than in an undivided population. As a result, a subdivided population can be more resistant to Muller’s ratchet than an undivided population of an equal overall size despite the fact that population subdivision accelerates Muller’s ratchet in the absence of HGT owing to the enhanced effect of genetic drift. Put differently, to maintain genomic information in the face of deleterious mutations and genetic drift, it is advantageous to partition individuals into multiple (smaller) subpopulations and let them “cross-reference” each other’s genetic information through recombination rather than collect all individuals in one population and thereby maximize the efficacy of natural selection. Note, however, that although population subdivision can be beneficial, it is not necessary for HGT to prevent Muller’s ratchet.

How do these findings relate to the previous population genetics studies on the advantage of recombination? Previous studies have shown that recombination is beneficial (compared with no recombination) when a population suffers from the reduced efficacy of natural selection because of negative linkage disequilibrium (*i.e.*, biased associations on chromosomes between beneficial and deleterious alleles) which arises from genetic drift (the Hill-Robertson effect) or synergetic epistasis (Hill and Robertson 1966; Felsenstein 1974; Feldman *et al.* 1980; Kondrashov 1988, 1993; Otto and Lenormand 2002). Our present results are consistent with these findings because they show that the reduction in the rate of mutation accumulation caused by HGT is greater in a subdivided population than in an undivided population (Figure 8, A and B). Indeed, population subdivision causes negative linkage disequilibrium in many pairs of loci in the least-loaded classes at the entire population level because the least-loaded classes in different subpopulations have mutations in different loci (this can be seen from the fact that *H_T_* > 0 and *H_S_* ≈ 0 when *D*_pop_ ≈ 0). In addition, the results show that the rate of mutation accumulation itself can be lower in a subdivided population than in an undivided population, despite the disadvantage of population subdivision due to enhanced drift. This conclusion does not immediately follow from the previous findings because the fact that negative linkage disequilibrium increases the benefit of recombination (compared with no recombination) does not necessarily mean that negative linkage disequilibrium is beneficial in preventing the accumulation of mutations in the presence of recombination.

Evolutionary maintenance of genomic information based on population subdivision and recombination hinges on the fact that genetic exchange between cells happens via a shared pool of eDNA. On the one hand, this implies that the indirectness of HGT might actually be an asset rather than a drawback for prokaryotes because HGT provides the possibility of exchange between physically separated subpopulations that accumulate different mutations.

On the other hand, indirect recombination imposes strict conditions under which population subdivision is advantageous in terms of Muller’s ratchet, requiring restricted migration between populations and rapid transport of eDNA molecules. Although at present it is difficult to judge whether such conditions are fulfilled in natural environments, the available data seem to suggest soils as an environment that is conducive to these processes. The distribution of bacteria in natural soils is “patchy,” *i.e.*, bacteria typically occur as small colonies separated from each other, and their locations are restricted by the availability of soil micro-pores, water, and organic substances (Foster 1988; Stotzky 1989; Grundmann 2004; Young *et al.* 2008; Vos *et al.* 2013). Moreover, bacterial motility can be highly restricted by water availability (Dechesne *et al.* 2010). In addition, soil bacteria display high levels of genetic diversity within local populations (Grundmann 2004). For example, *Agrobacterium* biovar 1 and *Nitrobacter*-like bacteria within minute soil samples (<1 mm in diameter) display genetic divergence as great as that between strains sampled from different geographical areas (Grundmann and Normand 2000; Vogel *et al.* 2003, although *Agrobacterium* biovar 1 is not known to be naturally competent, this bacterium readily undergoes homologous recombination; Costechareyre *et al.* 2009). Also, genomes of *Pseudomonas stutzeri* display extremely high gene diversity (*H_T_* > 0.8) and high frequency of null alleles (>80% of the examined strains fail to exhibit the activity of at least one of the 20 enzymes examined; Rius *et al.* 2001). Taken together, these findings suggest that bacterial populations in unsaturated soils are strongly subdivided and genetically heterogeneous within local environments. This population structure is compatible with a situation in which HGT and population subdivision jointly prevent the operation of Muller’s ratchet (if eDNA transport is sufficiently rapid as well).

Moreover, eDNA in soils becomes highly stable against hydrolysis by nucleases when it is absorbed to clay minerals, sand particles, and humic acids without losing the ability to transform competent bacteria (Vries and Wackernagel 2005; Nielsen *et al.* 2007; Pietramellara *et al.* 2009). Natural transformation assays indicate that DNA added to nonsterile soils retains transformation ability for 3−15 days (Gallori *et al.* 1994; Sikorski *et al.* 1998). In addition, eDNA can be transported within unsaturated soils through water capillarity or leaching (Ceccherini *et al.* 2007; Pote *et al.* 2010). Although the difference between bacteria and eDNA in terms of transport efficiency within soils remains to be evaluated, the obvious size difference suggests that eDNA is more readily transported than bacteria. Taken together, these findings suggest soil prokaryote systems as a prime candidate for a system in which HGT and population subdivision could be important for the long-term maintenance of genomic information in the face of Muller’s ratchet. Furthermore, it appears plausible that both the molecular machinery of natural transformation and GTAs evolved, at least in part, as adaptations facilitating HGT and overcoming the limitations on HGT imposed by the aforementioned conditions, *e.g.*, GTAs can increase the stability of DNA emitted from the cell.

Is Muller’s ratchet an important factor of evolution in prokaryotes? Its role can be questioned because many bacteria have large population sizes in which the extent of stochasticity is limited and that are accordingly immune to Muller’s ratchet (but see also the discussion of soil bacteria above in this section). However, bacteria are also known to go through frequent population bottlenecks under stress conditions (Levin *et al.* 2000; Aertsen and Michiels 2004; Wolf *et al.* 2005), and it is under these conditions that Muller’s ratchet and mechanisms that can overcome it could become relevant. Indeed, competence has been shown to increase stress resistance in bacteria (Engelmoer and Rozen 2011) and induction of the competence regulons is an important part of bacterial stress response (Claverys *et al.* 2006; Prudhomme *et al.* 2006). Although the biology of the GTAs is not nearly as well understood as that of competence and transformation, stress-induced prophage replication that is likely to potentiate HGT has been demonstrated (Garcia-Russell *et al.* 2009).

To conclude, the results of the modeling study described here indicate that HGT can prevent the operation of Muller’s ratchet, and this effect can be enhanced by population subdivision. Thus, through preventing the action of Muller’s ratchet, HGT could be a necessary condition for the long-term survival of prokaryotic populations. The benefits of HGT for prokaryotes certainly are not limited to the prevention of Muller’s ratchet and additionally include acquisition of novel genes and functions (see, *e.g.*, Levin and Cornejo 2009; Wylie *et al.* 2010). Jointly, these advantages could be the driving forces behind the evolution of dedicated mechanisms for HGT.

## Supplementary Material

Supporting Information

## References

[bib1] Aertsen A. Michiels C. W., 2004 Stress and how bacteria cope with death and survival. Crit. Rev. Microbiol. 30: 263–273 15646400 10.1080/10408410490884757

[bib2] Aravind L. Tatusov R. L. Wolf Y. I. Walker D. R. Koonin E. V., 1998 Evidence for massive gene exchange between archaeal and bacterial hyperthermophiles. Trends Genet. 14: 442–444 9825671 10.1016/s0168-9525(98)01553-4

[bib3] Bell G., 1988 Recombination and the immortality of the germ line. J. Evol. Biol. 1: 67–82

[bib4] Bernstein H. Hopf F. A. Michod R. E., 1988 Is meiotic recombination an adaptation for repairing DNA, producing genetic variation, or both? pp. 139–160 in The Evolution of Sex, edited by Michod R. E. Levin B. R. Sinauer Associates, Sunderland, MA

[bib5] Blahna M. T. Zalewski C. A. Reuer J. Kahlmeter G. Foxman B., 2006 The role of horizontal gene transfer in the spread of trimethoprim-sulfamethoxazole resistance among uropathogenic *Escherichia coli* in Europe and Canada. J. Antimicrob. Chemother. 57: 666–672 16464890 10.1093/jac/dkl020

[bib6] Brigulla M. Wackernagel W., 2010 Molecular aspects of gene transfer and foreign DNA acquisition in prokaryotes with regard to safety issues. Appl. Microbiol. Biotechnol. 86: 1027–1041 20191269 10.1007/s00253-010-2489-3

[bib7] Brochier-Armanet C. Forterre P., 2007 Widespread distribution of archaeal reverse gyrase in thermophilic bacteria suggests a complex history of vertical inheritance and lateral gene transfers. Archaea 2: 83–93 17350929 10.1155/2006/582916 PMC2686386

[bib8] Burke C. Steinberg P. Rusch D. Kjelleberg S. Thomas T., 2011 Bacterial community assembly based on functional genes rather than species. Proc. Natl. Acad. Sci. USA 108: 14288–14293 21825123 10.1073/pnas.1101591108 PMC3161577

[bib9] Ceccherini M. T. Ascher J. Pietramellara G. Vogel T. M. Nannipieri P., 2007 Vertical advection of extracellular DNA by water capillarity in soil columns. Soil Biol. Biochem. 39: 158–163

[bib10] Charlesworth B. Charlesworth D., 1997 Rapid fixation of deleterious alleles can be caused by Muller’s ratchet. Genet. Res. 70: 63–73 9369098 10.1017/s0016672397002899

[bib11] Charlesworth D. Morgan M. T. Charlesworth B., 1993 Mutation accumulation in finite outbreeding and inbreeding populations. Genet. Res. 61: 39–56

[bib12] Claverys J. P. Prudhomme M. Martin B., 2006 Induction of competence regulons as a general response to stress in gram-positive bacteria. Annu. Rev. Microbiol. 60: 451–475 16771651 10.1146/annurev.micro.60.080805.142139

[bib13] Claverys J. P. Martin B. Polard P., 2009 The genetic transformation machinery: composition, localization, and mechanism. FEMS Microbiol. Rev. 33: 643–656 19228200 10.1111/j.1574-6976.2009.00164.x

[bib14] Combadao J. Campos P. R. Dionisio F. Gordo I., 2007 Small-world networks decrease the speed of Muller’s ratchet. Genet. Res. 89: 7–18 17517155 10.1017/S0016672307008658

[bib15] Cooper T. F., 2007 Recombination speeds adaptation by reducing competition between beneficial mutations in populations of Escherichia coli. PLoS Biol. 5: e225 17713986 10.1371/journal.pbio.0050225 PMC1950772

[bib16] Costechareyre D. Bertolla F. Nesme X., 2009 Homologous recombination in Agrobacterium: potential implications for the genomic species concept in bacteria. Mol. Biol. Evol. 26: 167–176 18936442 10.1093/molbev/msn236

[bib17] Dagan T. Artzy-Randrup Y. Martin W., 2008 Modular networks and cumulative impact of lateral transfer in prokaryote genome evolution. Proc. Natl. Acad. Sci. USA 105: 10039–10044 18632554 10.1073/pnas.0800679105 PMC2474566

[bib18] Dechesne A. Wang G. Gulez G. Or D. Smets B. F., 2010 Hydration-controlled bacterial motility and dispersal on surfaces. Proc. Natl. Acad. Sci. USA 107: 14369–14372 20660312 10.1073/pnas.1008392107 PMC2922541

[bib19] Dubnau D., 1999 DNA uptake in bacteria. Annu. Rev. Microbiol. 53: 217–244 10547691 10.1146/annurev.micro.53.1.217

[bib20] Engelmoer D. J. Rozen D. E., 2011 Competence increases survival during stress in *Streptococcus pneumoniae*. Evolution 65: 3475–3485 22133219 10.1111/j.1558-5646.2011.01402.x

[bib21] Eppley J. M. Tyson G. W. Getz W. M. Banfield J. F., 2007 Genetic exchange across a species boundary in the archaeal genus ferroplasma. Genetics 177: 407–416 17603112 10.1534/genetics.107.072892 PMC2013692

[bib22] Feil E. J. Holmes E. C. Bessen D. E. Chan M. S. Day N. P., 2001 Recombination within natural populations of pathogenic bacteria: short-term empirical estimates and long-term phylogenetic consequences. Proc. Natl. Acad. Sci. USA 98: 182–187 11136255 10.1073/pnas.98.1.182 PMC14565

[bib23] Feil E. J. Smith J. M. Enright M. C. Spratt B. G., 2000 Estimating recombinational parameters in *Streptococcus pneumoniae* from multilocus sequence typing data. Genetics 154: 1439–1450 10747043 10.1093/genetics/154.4.1439 PMC1461021

[bib24] Feldman M. W. Christiansen F. B. Brooks L. D., 1980 Evolution of recombination in a constant environment. Proc. Natl. Acad. Sci. USA 77: 4838–4841 16592864 10.1073/pnas.77.8.4838 PMC349943

[bib25] Felsenstein J., 1974 The evolutionary advantage of recombination. Genetics 78: 737–756 4448362 10.1093/genetics/78.2.737 PMC1213231

[bib26] Foster R. C., 1988 Microenvironments of soil microorganisms. Biol. Fertil. Soils 6: 189–203

[bib27] Gallori E. Bazzicalupo M. Dal Canto L. Fani R. Nannipieri P., 1994 Transformation of *Bacillus subtilis* by DNA bound on clay in non-sterile soil. FEMS Microbiol. Ecol. 15: 119–126

[bib28] Garcia-Russell N. Elrod B. Dominguez K., 2009 Stress-induced prophage DNA replication in *Salmonella enterica* serovar Typhimurium. Infect. Genet. Evol. 9: 889–895 19501196 10.1016/j.meegid.2009.05.017

[bib29] Gogarten J. P. Doolittle W. F. Lawrence J. G., 2002 Prokaryotic evolution in light of gene transfer. Mol. Biol. Evol. 19: 2226–2238 12446813 10.1093/oxfordjournals.molbev.a004046

[bib30] Goyal S. Balick D. J. Jerison E. R. Neher R. A. Shraiman B. I., 2012 Dynamic mutation-selection balance as an evolutionary attractor. Genetics 191: 1309–1319 22661327 10.1534/genetics.112.141291 PMC3416009

[bib31] Grundmann G. L., 2004 Spatial scales of soil bacterial diversity--the size of a clone. FEMS Microbiol. Ecol. 48: 119–127 19712395 10.1016/j.femsec.2004.01.010

[bib32] Grundmann G. L. Normand P., 2000 Microscale diversity of the genus Nitrobacter in soil on the basis of analysis of genes encoding rRNA. Appl. Environ. Microbiol. 66: 4543–4546 11010914 10.1128/aem.66.10.4543-4546.2000 PMC92340

[bib33] Haigh J., 1978 The accumulation of deleterious genes in a population—Muller’s ratchet. Theor. Popul. Biol. 14: 251–267 746491 10.1016/0040-5809(78)90027-8

[bib34] Higgins K. Lynch M., 2001 Metapopulation extinction caused by mutation accumulation. Proc. Natl. Acad. Sci. USA 98: 2928–2933 11226343 10.1073/pnas.031358898 PMC30242

[bib35] Hill W. G. Robertson A., 1966 The effect of linkage on limits to artificial selection. Genet. Res. 8: 269–294 5980116

[bib36] Hulter N. Wackernagel W., 2008 Double illegitimate recombination events integrate DNA segments through two different mechanisms during natural transformation of *Acinetobacter baylyi*. Mol. Microbiol. 67: 984–995 18194157 10.1111/j.1365-2958.2007.06096.x

[bib37] Johnsborg O. Eldholm V. Havarstein L. S., 2007 Natural genetic transformation: prevalence, mechanisms and function. Res. Microbiol. 158: 767–778 17997281 10.1016/j.resmic.2007.09.004

[bib38] Johnston C. Martin B. Granadel C. Polard P. Claverys J. P., 2013 Programmed protection of foreign DNA from restriction allows pathogenicity island exchange during pneumococcal transformation. PLoS Pathog. 9: e1003178 23459610 10.1371/journal.ppat.1003178 PMC3573125

[bib39] Keightley P. D. Otto S. P., 2006 Interference among deleterious mutations favours sex and recombination in finite populations. Nature 443: 89–92 16957730 10.1038/nature05049

[bib40] Kelly B. G. Vespermann A. Bolton D. J., 2009 Horizontal gene transfer of virulence determinants in selected bacterial foodborne pathogens. Food Chem. Toxicol. 47: 969–977 18420327 10.1016/j.fct.2008.02.007

[bib41] Komatsu S., 1980 *Overstepping Biology* (“*Hamidashi Seibutsugaku*”) (*in Japanese*). Heibonsha, Tokyo

[bib42] Kondrashov A. S., 1988 Deleterious mutations and the evolution of sexual reproduction. Nature 336: 435–440 3057385 10.1038/336435a0

[bib43] Kondrashov A. S., 1993 Classification of hypotheses on the advantage of amphimixis. J. Hered. 84: 372–387 8409359 10.1093/oxfordjournals.jhered.a111358

[bib44] Koonin E. V., 2011 The Logic of Chance. FT Press Science, Upper Saddle River, NJ

[bib45] Koonin E. V. Makarova K. S. Aravind L., 2001 Horizontal gene transfer in prokaryotes: quantification and classification. Annu. Rev. Microbiol. 55: 709–742 11544372 10.1146/annurev.micro.55.1.709 PMC4781227

[bib46] Lang A. S. Beatty J. T., 2007 Importance of widespread gene transfer agent genes in alpha-proteobacteria. Trends Microbiol. 15: 54–62 17184993 10.1016/j.tim.2006.12.001

[bib47] Lang A. S. Zhaxybayeva O. Beatty J. T., 2012 Gene transfer agents: phage-like elements of genetic exchange. Nat. Rev. Microbiol. 10: 472–482 22683880 10.1038/nrmicro2802 PMC3626599

[bib48] Lerat E. Daubin V. Ochman H. Moran N. A., 2005 Evolutionary origins of genomic repertoires in bacteria. PLoS Biol. 3: e130 15799709 10.1371/journal.pbio.0030130 PMC1073693

[bib49] Levin B. R., 1988 The evolution of sex in bacteria, pp. 194–211 in The Evolution of Sex, edited by Michod R. E. Levin B. R. Sinauer Associates, Sunderland, MA

[bib50] Levin B. R. Cornejo O. E., 2009 The population and evolutionary dynamics of homologous gene recombination in bacterial populations. PLoS Genet. 5: e1000601 19680442 10.1371/journal.pgen.1000601 PMC2717328

[bib51] Levin B. R. Perrot V. Walker N., 2000 Compensatory mutations, antibiotic resistance and the population genetics of adaptive evolution in bacteria. Genetics 154: 985–997 10757748 10.1093/genetics/154.3.985 PMC1460977

[bib52] Lorenz M. G. Wackernagel W., 1994 Bacterial gene transfer by natural genetic transformation in the environment. Microbiol. Rev. 58: 563–602 7968924 10.1128/mr.58.3.563-602.1994 PMC372978

[bib53] Lynch M. Burger R. Butcher D. Gabriel W., 1993 The mutational meltdown in asexual populations. J. Hered. 84: 339–344 8409355 10.1093/oxfordjournals.jhered.a111354

[bib54] Majewski J. Cohan F. M., 1999 DNA sequence similarity requirements for interspecific recombination in Bacillus. Genetics 153: 1525–1533 10581263 10.1093/genetics/153.4.1525 PMC1460850

[bib55] Maslov S. Krishna S. Pang T. Y. Sneppen K., 2009 Toolbox model of evolution of prokaryotic metabolic networks and their regulation. Proc. Natl. Acad. Sci. USA 106: 9743–9748 19482938 10.1073/pnas.0903206106 PMC2701025

[bib56] Maynard Smith J., 1978 The Evolution of Sex. Cambridge University Press, Cambridge, UK

[bib57] Maynard Smith J., 1998 Evolutionary Genetics. Oxford University Press, New York

[bib58] McCutcheon J. P. Moran N. A., 2012 Extreme genome reduction in symbiotic bacteria. Nat. Rev. Microbiol. 10: 13–26 22064560 10.1038/nrmicro2670

[bib59] McDaniel L. D. Young E. Delaney J. Ruhnau F. Ritchie K. B., 2010 High frequency of horizontal gene transfer in the oceans. Science 330: 50 20929803 10.1126/science.1192243

[bib60] Merhej V. Raoult D., 2011 Rickettsial evolution in the light of comparative genomics. Biol. Rev. Camb. Philos. Soc. 86: 379–405 20716256 10.1111/j.1469-185X.2010.00151.x

[bib61] Michod R. E. Levin B. R. (Editors), 1988 The Evolution of Sex. Sinauer Associates, Sunderland, MA

[bib62] Moran N. A., 1996 Accelerated evolution and Muller’s ratchet in endosymbiotic bacteria. Proc. Natl. Acad. Sci. USA 93: 2873–2878 8610134 10.1073/pnas.93.7.2873 PMC39726

[bib63] Mulkidjanian A. Y. Koonin E. V. Makarova K. S. Mekhedov S. L. Sorokin A., 2006 The cyanobacterial genome core and the origin of photosynthesis. Proc. Natl. Acad. Sci. USA 103: 13126–13131 16924101 10.1073/pnas.0605709103 PMC1551899

[bib64] Muller H. J., 1964 The relation of recombination to mutational advance. Mutat. Res. 106: 2–9 14195748 10.1016/0027-5107(64)90047-8

[bib65] Neher R. A. Shraiman B. I., 2012 Fluctuations of fitness distributions and the rate of Muller’s ratchet. Genetics 191: 1283–1293 22649084 10.1534/genetics.112.141325 PMC3416007

[bib66] Nei M., 1987 Molecular Evolutionary Genetics. Columbia University Press, New York

[bib67] Nielsen K. M. Johnsen P. J. Bensasson D. Daffonchio D., 2007 Release and persistence of extracellular DNA in the environment. Environ. Biosafety Res. 6: 37–53 17961479 10.1051/ebr:2007031

[bib68] Novichkov P. S. Wolf Y. I. Dubchak I. Koonin E. V., 2009 Trends in prokaryotic evolution revealed by comparison of closely related bacterial and archaeal genomes. J. Bacteriol. 191: 65–73 18978059 10.1128/JB.01237-08 PMC2612427

[bib69] Ochman H. Lawrence J. G. Groisman E. A., 2000 Lateral gene transfer and the nature of bacterial innovation. Nature 405: 299–304 10830951 10.1038/35012500

[bib70] Otto S. P. Lenormand T., 2002 Resolving the paradox of sex and recombination. Nat. Rev. Genet. 3: 252–261 11967550 10.1038/nrg761

[bib71] Pál C. Papp B. Lercher M. J., 2005 Adaptive evolution of bacterial metabolic networks by horizontal gene transfer. Nat. Genet. 37: 1372–1375 16311593 10.1038/ng1686

[bib72] Pamilo P. Nei M. Li W.-H., 1987 Accumulation of mutations in sexual and asexual populations. Genet. Res. 49: 135–146 3596234 10.1017/s0016672300026938

[bib73] Pietramellara G. Ascher J. Borgogni F. Ceccherini M. T. Guerri G., 2009 Extracellular DNA in soil and sediment: fate and ecological relevance. Biol. Fertil. Soils 45: 219–235

[bib74] Pote J. Teresa Ceccherini M. Rosselli W. Wildi W. Simonet P., 2010 Leaching and transformability of transgenic DNA in unsaturated soil columns. Ecotoxicol. Environ. Saf. 73: 67–72 19828198 10.1016/j.ecoenv.2009.09.009

[bib75] Prudhomme M. Attaiech L. Sanchez G. Martin B. Claverys J. P., 2006 Antibiotic stress induces genetic transformability in the human pathogen *Streptococcus pneumoniae*. Science 313: 89–92 16825569 10.1126/science.1127912

[bib76] Redfield R. J., 1988 Evolution of bacterial transformation: is sex with dead cells ever better than no sex at all? Genetics 119: 213–221 3396864 10.1093/genetics/119.1.213 PMC1203342

[bib77] Redfield R. J., 2001 Do bacteria have sex? Nat. Rev. Genet. 2: 634–639 11483988 10.1038/35084593

[bib78] Redfield R. J. Schrag M. R. Dean A. M., 1997 The evolution of bacterial transformation: sex with poor relations. Genetics 146: 27–38 9135998 10.1093/genetics/146.1.27 PMC1207942

[bib79] Rius N. Fuste M. C. Guasp C. Lalucat J. Loren J. G., 2001 Clonal population structure of *Pseudomonas stutzeri*, a species with exceptional genetic diversity. J. Bacteriol. 183: 736–744 11133969 10.1128/JB.183.2.736-744.2001 PMC94931

[bib80] Rouzine I. M. Brunet E. Wilke C. O., 2008 The traveling-wave approach to asexual evolution: Muller’s ratchet and speed of adaptation. Theor. Popul. Biol. 73: 24–46 18023832 10.1016/j.tpb.2007.10.004 PMC2246079

[bib81] Shapiro B. J. Friedman J. Cordero O. X. Preheim S. P. Timberlake S. C., 2012 Population genomics of early events in the ecological differentiation of bacteria. Science 336: 48–51 22491847 10.1126/science.1218198 PMC3337212

[bib82] Sikorski J. Graupner S. Lorenz M. G. Wackernagel W., 1998 Natural genetic transformation of *Pseudomonas stutzeri* in a non-sterile soil. Microbiology 144: 569–576 9493393 10.1099/00221287-144-2-569

[bib83] Stotzky G., 1989 Gene transfer among bacteria in soil, pp. 165–222 in Gene Transfer in the Environment. edited by Levy S. B. Miller R. V. McGraw-Hill, New York

[bib84] Thomas C. M. Nielsen K. M., 2005 Mechanisms of, and barriers to, horizontal gene transfer between bacteria. Nat. Rev. Microbiol. 3: 711–721 16138099 10.1038/nrmicro1234

[bib85] Treangen T. J. Rocha E. P., 2011 Horizontal transfer, not duplication, drives the expansion of protein families in prokaryotes. PLoS Genet. 7: e1001284 21298028 10.1371/journal.pgen.1001284 PMC3029252

[bib86] Vogel J. Normand P. Thioulouse J. Nesme X. Grundmann G. L., 2003 Relationship between spatial and genetic distance in *Agrobacterium* spp. in 1 cubic centimeter of soil. Appl. Environ. Microbiol. 69: 1482–1487 12620832 10.1128/AEM.69.3.1482-1487.2003 PMC150114

[bib87] Vos M., 2009 Why do bacteria engage in homologous recombination? Trends Microbiol. 17: 226–232 19464181 10.1016/j.tim.2009.03.001

[bib88] Vos M. Didelot X., 2009 A comparison of homologous recombination rates in bacteria and archaea. ISME J. 3: 199–208 18830278 10.1038/ismej.2008.93

[bib89] Vos M. Wolf A. B. Jennings S. J. Kowalchuk G. A., 2013 Micro-scale determinants of bacterial diversity in soil. FEMS Microbiol. Rev. 37: 936–954 23550883 10.1111/1574-6976.12023

[bib90] Vries J. Wackernagel W., 2005 Microbial horizontal gene transfer and the DNA release from transgenic crop plants. Plant Soil 266: 91–104

[bib91] Watt V. M. Ingles C. J. Urdea M. S. Rutter W. J., 1985 Homology requirements for recombination in *Escherichia coli*. Proc. Natl. Acad. Sci. USA 82: 4768–4772 3161076 10.1073/pnas.82.14.4768 PMC390986

[bib92] Wolf D. M. Vazirani V. V. Arkin A. P., 2005 Diversity in times of adversity: probabilistic strategies in microbial survival games. J. Theor. Biol. 234: 227–253 15757681 10.1016/j.jtbi.2004.11.020

[bib93] Wylie C. S. Trout A. D. Kessler D. A. Levine H., 2010 Optimal strategy for competence differentiation in bacteria. PLoS Genet. 6: e1001108 20838595 10.1371/journal.pgen.1001108 PMC2936531

[bib94] Yahara K. Kawai M. Furuta Y. Takahashi N. Handa N., 2012 Genome-wide survey of mutual homologous recombination in a highly sexual bacterial species. Genome Biol. Evol. 4: 628–640 22534164 10.1093/gbe/evs043 PMC3381677

[bib95] Young I. M. Crawford J. W. Nunan N. Otten W. Spiers A., 2008 Microbial distribution in soils: physics and scaling, pp. 81–121 in Advances in Agronomy. edited by Donald L. S. Academic Press, San Diego

[bib96] Zafra O. Lamprecht-Grandio M. de Figueras C. G. Gonzalez-Pastor J. E., 2012 Extracellular DNA release by undomesticated *Bacillus subtilis* is regulated by early competence. PLoS ONE 7: e48716 23133654 10.1371/journal.pone.0048716 PMC3487849

