# Supplementary material for: Horizontal Gene Transfer Can Rescue Prokaryotes from Muller’s Ratchet: Benefit of DNA from Dead Cells and Population Subdivision
Source: G3 (Bethesda). 2013 Dec 17;4(2):325–39. doi: 10.1534/g3.113.009845 (PMC3931566; doi:10.1534/g3.113.009845)
Supplement: Supporting Information [file supp_4_2_325__index.html]

Horizontal Gene Transfer Can Rescue Prokaryotes from Muller’s Ratchet: Benefit of DNA from Dead Cells and Population Subdivision — Supporting Information 

# Horizontal Gene Transfer Can Rescue Prokaryotes from Muller’s Ratchet: Benefit of DNA from Dead Cells and Population Subdivision

## Supporting Information for Takeuchi, Kaneko, and Koonin, 2014

**Files in this Data Supplement:**

- File S1 - Calculation of the average number of deleterious mutations per *l* loci in the eDNA pool. (PDF, 570 KB)
