# Supplementary material for: Horizontal Gene Transfer Can Rescue Prokaryotes from Muller’s Ratchet: Benefit of DNA from Dead Cells and Population Subdivision
Source: G3 (Bethesda). 2013 Dec 17;4(2):325–39. doi: 10.1534/g3.113.009845 (PMC3931566; doi:10.1534/g3.113.009845)
Supplement: Supporting Information [file supp_g3.113.009845_009845SI.pdf]

G3 · Genes | Genomes | Genetics

Horizontal Gene Transfer Can Rescue Prokaryotes from Muller's Ratchet: Benefit of DNA from Dead Cells and Population Subdivision

Nobuto Takeuchi[*§], Kunihiko Kaneko[§], and Eugene V. Koonin[*]

[*]National Center for Biotechnology Information, National Library of Medicine, National Institutes of Health, Bethesda, MD 20894

[§] Department of Basic Science, Graduate School of Arts and Sciences, University of Tokyo, Tokyo 153-8902, Japan

Correspondence: Nobuto Takeuchi (takeuchi.nobuto@gmail.com). The University of Tokyo, KOMABA, Graduate School of Arts and Sciences, Department of Basic Science, Komaba 3-8-1, Meguro-ku, Tokyo 153-8902, Japan.

DOI: 10.1534/g3.113.009845

File S1

Calculation of the average number of deleterious mutations per l loci in the eDNA pool

Below, we derive the distribution of deleterious mutations per l loci in the eDNA pool, assuming that the population size N is very large (and so $m_{\mathrm{LLC}} = 0$) and eDNA turnover is very rapid ($d_{\mathrm{eDNA}} = 1$). Because of rapid eDNA turnover, this distribution is identical to the distribution of deleterious mutations in the genomes contributed by dying individuals in one generation. As described in the main text, an individual with i mutations contributes its genome to the eDNA pool with a probability proportional to $D_i = 1 - (1 - D_0)(1 - s)^i$. The fraction G_i of genomes with i mutations that are released into the eDNA pool in one generation is $G_i = D_i n_i / \sum_{j=0}^{\infty} D_j n_j$. According to Haigh (1978), n_i is approximated by

$$n_i \approx \frac{1}{i!}\left(\frac{U}{s}\right)^i e^{-\frac{U}{s}} N .$$

Using this approximation, $\sum_i D_i n_i$ is calculated as follows:

$$\begin{aligned}
\sum_{i=0}^{\infty} D_i n_i &\approx \sum_{i=0}^{\infty} \left[1 - (1 - D_0)(1 - s)^i\right] \frac{1}{i!}\left(\frac{U}{s}\right)^i e^{-\frac{U}{s}} \\
&= \left\{ \sum_{i=0}^{\infty} \frac{1}{i!}\left(\frac{U}{s}\right)^i - (1 - D_0) \sum_{i=0}^{\infty} \frac{1}{i!}\left[\frac{(1-s)U}{s}\right]^i \right\} e^{-\frac{U}{s}} \\
&= \left\{ e^{\frac{U}{s}} - (1 - D_0) e^{\frac{(1-s)U}{s}} \right\} e^{-\frac{U}{s}} \\
&= 1 - (1 - D_0) e^{-U}
\end{aligned}$$

Therefore,

$$G_i = \frac{1 - (1 - D_0)(1 - s)^i}{1 - (1 - D_0) e^{-U}} \frac{1}{i!}\left(\frac{U}{s}\right)^i e^{-\frac{U}{s}} .$$

A similar calculation yields the average $\bar{G}_i = \sum_{i=0}^{\infty} i G_i$ as follows:

$$\bar{G}_i = \frac{U}{s} + \frac{(1 - D_0) U e^{-U}}{1 - (1 - D_0) e^{-U}} .$$

Because $d\bar{G}_i / dD_0 = -U / \left[1 - (1 - D_0) e^{-U}\right]^2$, $\bar{G}_i$ monotonically decreases with the increase of D_0. Thus, setting $D_0 = 0$ maximizes $\bar{G}_i$ as mentioned in the main text. When $D_0 = 0$, $\bar{G}_i = U / s + U e^{-U} / \left(1 - e^{-U}\right)$. Because $U << 1$, $\bar{G}_i \approx U / s + 1$ (Redfield *et al.* 1997).

Calculation of the probability P_{10} that an HGT event transforms one but the least-loaded class into the least-loaded class and the probability P_{01} that an HGT event introduces at least one mutation in the least-loaded class

Below, we derive P_{10}, again assuming a very large population and rapid eDNA turnover. P_{10} can be decomposed into two parts: the probability (l^{-1}) that HGT occurs to the locus in which one but the least-loaded class has a mutation that is not carried by the least-loaded class; and the probability that an allele randomly drawn from the eDNA pool does not contain this mutation (Y_0). Y_j is the fraction of alleles with j mutations for any given locus in the eDNA pool. Y_j can be calculated as follows. When an individual with i mutations dies, it releases alleles that carry 0 to i mutations into the eDNA pool. Because mutations are randomly distributed across l loci, the number of mutations in one locus is binomially distributed for different individuals. Thus, $Y_j = y_j / \sum_{i=0}^{\infty} y_i$ where

$$y_j = \sum_{i=j}^{\infty} \binom{i}{j} \left(l^{-1}\right)^j \left(1-l^{-1}\right)^{i-j} D_i n_i$$

Using the approximation for n_i used in the previous section, y_i is calculated as follows:

$$\begin{aligned}
y_j &= \sum_{i=j}^{\infty} \binom{i}{j} \left(l^{-1}\right)^j \left(1-l^{-1}\right)^{i-j} \left[1-\left(1-D_0\right)\left(1-s\right)^i\right] \frac{1}{i!} \left(U/s\right)^i e^{-U/s} \\
&= \left(l^{-1}\right)^j e^{-U/s} \sum_{i=j}^{\infty} \frac{1}{i!} \binom{i}{j} \left(1-l^{-1}\right)^{i-j} \left[1-\left(1-D_0\right)\left(1-s\right)^i\right] \left(U/s\right)^i \\
&= \left(l^{-1}\right)^j e^{-U/s} \sum_{i=j}^{\infty} \frac{1}{j!(i-j)!} \left(1-l^{-1}\right)^{i-j} \left[1-\left(1-D_0\right)\left(1-s\right)^i\right] \left(U/s\right)^i \\
&= \frac{1}{j!} \left(\frac{U}{ls}\right)^j e^{-U/s} \sum_{i=j}^{\infty} \frac{1}{(i-j)!} \left(1-l^{-1}\right)^{i-j} \left\{ \left(U/s\right)^{i-j} - \left(1-D_0\right)\left(1-s\right)^j \left[\left(1-s\right)U/s\right]^{i-j} \right\} \\
&= \frac{1}{j!} \left(\frac{U}{ls}\right)^j e^{-U/s} \sum_{i=0}^{\infty} \frac{1}{i!} \left(1-l^{-1}\right)^{i} \left\{ \left(U/s\right)^{i} - \left(1-D_0\right)\left(1-s\right)^j \left[\left(1-s\right)U/s\right]^{i} \right\} \\
&= \frac{1}{j!} \left(\frac{U}{ls}\right)^j e^{-U/s} \left[e^{\left(1-l^{-1}\right)U/s} - \left(1-D_0\right)\left(1-s\right)^j e^{\left(1-l^{-1}\right)\left(1-s\right)U/s} \right] \\
&= \frac{1}{j!} \left(\frac{U}{ls}\right)^j e^{-\frac{U}{ls}} \left[1 - \left(1-D_0\right)\left(1-s\right)^j e^{-\left(1-l^{-1}\right)U} \right].
\end{aligned}$$

A similar calculation yields $\sum_{j=0}^{\infty} y_j = 1-\left(1-D_0\right)e^{-U}$. Using these equations, when $D_0 = 0$, we obtain

$$Y_0 = \frac{e^{-\frac{U}{ls}} \left[1-e^{-\left(1-l^{-1}\right)U}\right]}{1-e^{-U}}.$$

Because $U \ll 1$ and $0 < l^{-1} \le 1$, Y_0 can be approximated by $\left(1-l^{-1}\right)e^{-\frac{U}{ls}}$. Thus, P_{10} is calculated as follows:

$$P_{10} = l^{-1} Y_0$$
$$\approx l^{-1}\left(1 - l^{-1}\right) e^{-\frac{U}{ls}}.$$

Next, we calculate the value of l that maximizes P_{10} ($\tilde{l}$) as follows. Let $\lambda = l^{-1}$. Differentiating P_{10} with respect to λ yields

$$dP_{10} / d\lambda = e^{-\lambda U/s}\left[\left(U/s\right) r^2 - \left(U/s - 2\right) r + 1\right]$$

Setting $dP_{10} / d\lambda = 0$ yields $\tilde{\lambda} = s/U + 1/2 \pm \sqrt{\left(s/U\right)^2 + 1/4}$ where $\tilde{\lambda} = \tilde{l}^{-1}$. When $\left(s/U\right)^2 << 1$, $\tilde{\lambda} \approx s/U + 1/2 \pm \left(1/2\right)\left[1 + 2\left(s/U\right)^2\right]$. Because $\lambda \leq 1$, we should choose

$$\tilde{l}^{-1} = \tilde{\lambda} = s/U + 1/2 - \sqrt{\left(s/U\right)^2 + 1/4}.$$

Likewise, P_{01} can be calculated as follows:

$$P_{01} = 1 - Y_0$$
$$\approx 1 - \left(1 - l^{-1}\right) e^{-\frac{U}{ls}}.$$

Dynamics of mutation fixation after the click of Muller's ratchet

As described in the main text (Figure 6), the speed of Muller's ratchet is nearly independent of the turnover rate of eDNA unless the characteristic lifetime of eDNA exceeds the generation time of organisms by orders of magnitude (in the absence of population subdivision). This is because the time between the click of Muller's ratchet and the subsequent fixation of a mutation is much longer than the generation time of individuals (Figure 7). In what follows, we give a more detailed explanation of this result, using a simple mathematical model.

Immediately after Muller's ratchet clicks, the least-loaded class contains multiple genotypes (i.e., $H_{m_{LLC}} > 0$). Let us consider separately each of these genotypes and the line of mutants descended from it. The population dynamics of each of these lines can be approximated by

$$\dot{x}_{m_{LLC}+k} = f_{m_{LLC}+k} x_{m_{LLC}+k} - U x_{m_{LLC}+k} + U x_{m_{LLC}+k-1} - \left\langle f \right\rangle x_{m_{LLC}+k}$$

where $x_{m_{\mathrm{LLC}}+k}$ is the population size of a line with $m_{\mathrm{LLC}} + k$ mutations, except for $k = 0$ (for $k = 0$, this approximation is invalid because the population dynamics of the least-loaded class is driven by genetic drift). This equation can be transformed into

$$\dot{x}_{m_{\mathrm{LLC}}+k} = -sk x_{m_{\mathrm{LLC}}+k} + U x_{m_{\mathrm{LLC}}+k-1}$$

by using $\left\langle f \right\rangle = f_{m_{LLC}} - U$ (which is obtained by assuming $\dot{n}_{m_{\mathrm{LLC}}} = 0$) and $f_m \approx 1 - ms$ (assuming $ms << 1$). This equation indicates that the extinction of a given line ($x_{m_{\mathrm{LLC}}+k-1} \to 0$) leads to the extinction of its descendant ($x_{m_{\mathrm{LLC}}+k} \to 0$). When, for a given value of k, every line goes extinct ($x_{m_{\mathrm{LLC}}+k} \to 0$), except for the one that descends from the least-loaded genotype that has fixed, $H_{m_{LLC}+k}$ reaches a steady-state level. When this extinction occurs for all values

of k, a mutation is fixed in the entire population—at this point, q_{eDNA} reaches zero (assuming fast eDNA turnover). In the slow ratchet regime, the population size of the least-loaded class ($n_{m_{\mathrm{LLC}}}$) is large, so that the timescale on which $x_{m_{\mathrm{LLC}}}$ decreases to zero is much slower than that on which $x_{m_{\mathrm{LLC}}+k}$ decreases to zero (i.e., $n_{m_{LLC}}^{-1} << (ks)^{-1}$). In this case, $x_{m_{\mathrm{LLC}}+k}$ will nearly synchronously drop to zero for all k values, so that the decrease of q_{eDNA} is synchronized with that of $H_{m_{LLC}}$. In the slow ratchet regime, $n_{m_{\mathrm{LLC}}}$ is small, so that the timescale on which $x_{m_{\mathrm{LLC}}}$ decreases to zero is as fast as the timescale on which $x_{m_{\mathrm{LLC}}+k}$ decreases to zero (i.e., $n_{m_{LLC}}^{-1} \geq (ks)^{-1}$). In this case, $x_{m_{\mathrm{LLC}}+k}$ sequentially decreases to zero for different values of k, so that the decrease of q_{eDNA} is delayed with respect to that of $H_{m_{LLC}}$, and it takes longer than s^{-1} generations for q_{eDNA} to reach zero because all mutant classes must go extinct. Since the value of s was set to 0.01 in all the simulations (for simplicity as described above), d_{eDNA} must be set substantially smaller than 0.01 to delay the decline of q_{eDNA} significantly. Since it seems unrealistic to consider a situation where $s >> 0.01$ in terms of Muller's ratchet, we can conclude that slow eDNA turnover by itself is unlikely to help HGT prevent the accumulation of mutations under realistic conditions.

$\Delta\bar{m}_{\mathrm{fix}} / \Delta t$ as a function of D_{pop} for various combinations of N and $s\bar{n}_{m_{\mathrm{LLC}}}$ values

The following figure supplements the data presented in Figure 8.

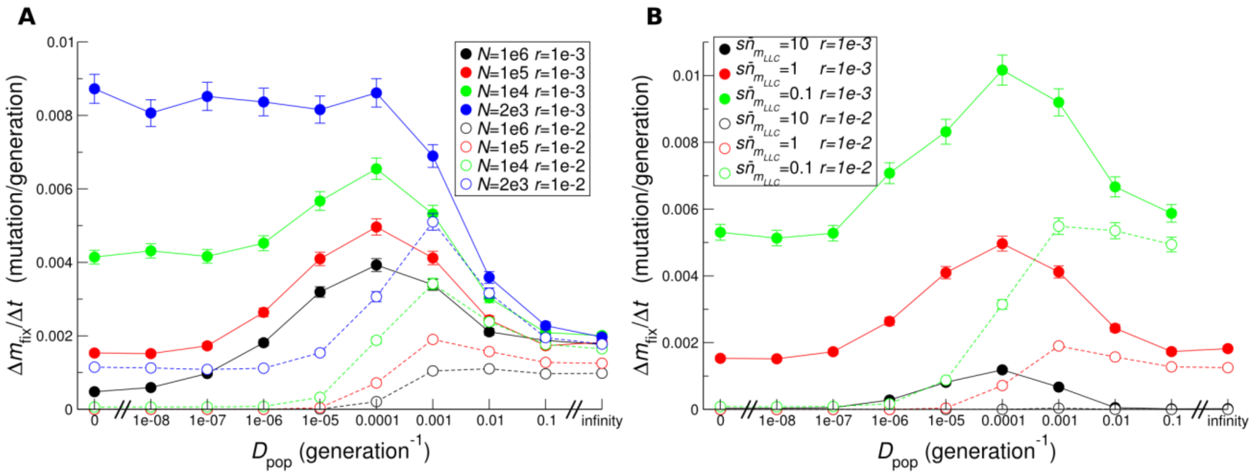

In A, the rate of mutation accumulation $\Delta\bar{m}_{\mathrm{fix}} / \Delta t$ is plotted as a function of population migration rate D_{pop} for various values of population sizes N and recombination rates r with $\bar{n}_{m_{\mathrm{LLC}}}$ fixed at unity (i.e., slow ratchet regime). In B, $\Delta\bar{m}_{\mathrm{fix}} / \Delta t$ is plotted as a function of D_{pop} for various values of $\bar{n}_{m_{\mathrm{LLC}}}$ and r with N fixed at 10^5.

REFERENCES

HAIGH, J., 1978 The accumulation of deleterious genes in a population—Muller's Ratchet. Theoretical Population Biology **14:** 251-267.

REDFIELD, R. J., M. R. SCHRAG and A. M. DEAN, 1997 The evolution of bacterial transformation: sex with poor relations. Genetics **146:** 27-38.
